# Acceptance and preferences for different nicotine substitute products to reduce tobacco smoking in people living with HIV: Results from an internal pilot study of a randomized trial

**DOI:** 10.18332/tid/218987

**Published:** 2026-05-26

**Authors:** Christof M. Schönenberger, Stephanie Campos Ochoa, Benjamin Speich, David Hans-Ulrich Haerry, Ellen Cart-Richter, David Jackson-Perry, Alissa Hutter, Loan Panettieri, Samuel Aggeler, Sandra E. Chaudron, Alexandra Calmy, Matthias Cavassini, Irene A. Abela, Katharina Kusejko, Huldrych F. Günthard, Johannes Nemeth, Andri Rauch, Gilles Wandeler, Patrick Schmid, Tamara Dörr, Bernard Surial, Aurélie Berthet, Reto Auer, Marcel P. Stoeckle, Niklaus Labhardt, Frédérique Chammartin, Matthias Briel, Alain Amstutz

**Affiliations:** 1 Division of Clinical Epidemiology, Department of Clinical Research, University Hospital Basel, University of Basel, Basel, Switzerland; 2 RETUNE-HIV Participant Advisory Board and Patient Organization ‘Positive Council’, Bern, Switzerland; 3 RETUNE-HIV Participant Advisory Board and Lausanne HIV-Community Council, University Hospital Lausanne, University of Lausanne, Lausanne, Switzerland; 4 RETUNE-HIV Participant Advisory Board and Infectious Diseases Service, University Hospital Lausanne, University of Lausanne, Lausanne, Switzerland; 5 Department of Infectious Diseases and Hospital Epidemiology, University Hospital Zurich, Zurich, Switzerland; 6 Institute of Medical Virology, University of Zurich, Zurich, Switzerland; 7 HIV Unit, Division of Infectious Diseases, University Hospital Geneva, University of Geneva, Geneva, Switzerland; 8 Division of Infectious Diseases, University Hospital Lausanne, University of Lausanne, Lausanne, Switzerland; 9 Department of Infectious Diseases, Inselspital, Bern University Hospital, University of Bern, Bern, Switzerland; 10 Division of Infectious Diseases, Infection Prevention and Travel Medicine, HOCH Health Ostschweiz, Cantonal Hospital St. Gallen, St. Gallen, Switzerland; 11 Center for Primary Care and Public Health, University of Lausanne, Lausanne, Switzerland; 12 Institute of Primary Health Care, University of Bern, Bern, Switzerland; 13 Division of Infectious Diseases, University Hospital Basel, University of Basel, Basel, Switzerland; 14 Department of Health Research Methods, Evidence, and Impact, McMaster University, Hamilton, Canada; 15 Population Health Sciences, Bristol Medical School, University of Bristol, Bristol, United Kingdom; 16 Oslo Centre for Biostatistics and Epidemiology, Oslo University Hospital, University of Oslo, Oslo, Norway

**Keywords:** TwiCs, HIV, smoking cessation, e-cigarettes, nicotine pouches

## Abstract

**INTRODUCTION:**

Tobacco smoking is an insufficiently addressed health burden among people living with HIV. The *Reduce tobacco use in people living with HIV in Switzerland* (RETUNE) trial tests the effectiveness of offering a menu of nicotine substitute products, including e-cigarettes, nicotine pouches, and nicotine patches, on tobacco smoking cessation rates. This a priori planned internal pilot aims to assess the trial processes, in particular the delivery of the intervention, and its acceptance by participants.

**METHODS:**

RETUNE is a pragmatic, randomized, multicenter trial using the ‘Trials within Cohorts’ (TwiCs) design embedded in the Swiss HIV Cohort Study. Participants are people living with HIV who are part of the cohort and smoke. Following the TwiCs design, participants can accept one of the offered intervention products or refuse all of them, after being randomized to the intervention group. This internal pilot study included the first 200 RETUNE participants randomized between February and September 2025. We report the acceptance rate and participants’ experiences with the intervention products, based on trial data and additional questionnaires.

**RESULTS:**

Of the 98 participants randomized to the intervention group, 53 accepted a nicotine substitute product while 45 declined the intervention. The majority chose e-cigarettes (31/53; 59%), one-third chose nicotine patches (17/53; 32%), and four participants chose nicotine pouches (4/53; 8%). The most common reason for declining all offered products was no interest in quitting tobacco smoking (34/53; 64%). Overall, 37/53 (70%) intervention participants completed the pilot study survey. Most (27/37; 73%) continued the initially chosen product; the remaining changed the product, the nicotine concentration, or the flavor; however, no one stopped the smoking cessation intervention entirely.

**CONCLUSIONS:**

The results of this internal pilot support the feasibility of the RETUNE trial. The observed acceptance rate was similar to our estimate. E-cigarettes were the preferred product. We are continuing recruitment for the RETUNE trial.

**CLINICAL TRIAL REGISTRATION:**

The study is registered on the official website of ClinicalTrials.gov

**IDENTIFIER:**

NCT06789692

## INTRODUCTION

In high-income settings, cardiovascular diseases and cancer have become the leading causes of death among people living with HIV (PLWH)^1,2^. Tobacco smoking is a major etiological factor for both diseases. High smoking prevalence among PLWH indicates a lack of effective smoking cessation interventions^3^.

Evidence from randomized clinical trials suggests that nicotine substitute products can improve quitting tobacco smoking and, therefore, reduce the associated health burden^4^. However, previous smoking cessation trials predominantly included people who were motivated to quit smoking and focused on testing a single nicotine substitution product^5,6^, which limits the clinical applicability of the results.

The *Reduce tobacco use in people living with HIV in Switzerland* (RETUNE) trial tests the effectiveness of offering a menu of nicotine substitute products, including e-cigarettes, nicotine pouches, and nicotine patches, on smoking cessation rate, to PLWH who smoke tobacco cigarettes regardless of their willingness to quit smoking (‘opt-out’ approach) (NCT06789692). RETUNE follows the Trials within Cohorts (TwiCs) design^7^. In TwiCs, participants first consent to the longitudinal data collection (cohort consent) and second, to be randomized into future trials (randomization consent). Participants who meet the eligibility criteria of a specific trial are then randomized. People in the control group are not informed about their allocation, while those randomized to the intervention group can accept or refuse the offered intervention, i.e. one of the products.

The objectives of this internal pilot study were to: 1) assess the acceptance rate of the offered intervention products among participants; 2) evaluate participants’ experiences with the intervention products in terms of satisfaction, handling, and side effects; and 3) refine the menu of offered products for the ongoing RETUNE trial.

## METHODS

The reporting of this manuscript follows the CONSORT statement extension for pilot studies^8^.

### RETUNE design

RETUNE is an ongoing, multicenter, pragmatic, 1:1 randomized, superiority clinical trial using the TwiCs design. Since 20 February 2025, we have been recruiting participants from the Swiss HIV Cohort Study (SHCS) at six participating hospitals (Basel, Zurich, St. Gallen, Bern, Geneva, Lausanne)^9^. The end of recruitment is estimated for December 2026.

The SHCS protocol (BASEC 2023-02080) and the RETUNE protocol, including the internal pilot study (BASEC 2024-02417), were approved by all involved ethics committees. All participants provided written cohort and randomization consent. The participants who accepted the intervention provided written informed consent.

Details on the trial design can be found in the protocol (Supplementary file Appendix 1). In brief, RETUNE includes participants aged ≥18 years, who signed the general SHCS randomization consent and smoke at least one tobacco cigarette per day. Pregnant women, as well as people who use e-cigarettes, nicotine pouches, or nicotine patches at trial start, are not eligible. The planned sample size of 972 participants was calculated under the assumption of a 50% acceptance rate of the offered intervention, i.e. participants selected an intervention product from the options provided. Eligibility is assessed automatically during routine cohort visits by an algorithm embedded in the SHCS database, which evaluates each participant’s current characteristics in real time. Eligible participants are then randomized by physicians using central randomization with minimization^10^. The physicians have no access to the random allocation sequence but are aware of the group assignment.

### RETUNE interventions

Participants randomized to the control group receive usual care and are not informed about the RETUNE trial. Participants randomized to the intervention will be offered a menu of different nicotine substitute products in addition to usual care: e-cigarettes (pod system OBY from Aspire^©^ with 1.2 ohm coils) and e-liquids (from Gaiatrend^©^; menthol and tobacco flavor; nicotine concentrations 3 , 6, and 16 mg/mL; all e-liquids had a ratio of propylene glycol to vegetal glycerin of 70:30), nicotine pouches [from Edelsnus^©^; menthol (20 mg/g) and mountain herbs (25 mg/g) flavor], and nicotine patches (from Nicotinell^©^; nicotine concentration 21, 14, and 7 mg/24 h). At inclusion, participants may select one of the offered products. Product changes are permitted at 4 weeks for nicotine patch users and at 8 weeks for users of e-cigarettes or nicotine pouches. At these time points, participants can request supplies or switch products by completing an online questionnaire. In case the questionnaire is not completed, the study team contacts participants by telephone. If participants cannot be reached, the same product as in the previous interval is being provided. At week 16, the study team again contacts participants to organize further supply. However, product changes are no longer possible at this point. In total, participants receive study products free of charge for 24 weeks. All participants receive an information brochure containing usage instructions, potential side effects, and procedures for requesting additional supplies.

### Pilot study design and participants

This internal pilot study was prespecified in the RETUNE protocol (Supplementary file Appendix 1). Following the concept of internal pilot studies, all participants included in this report will remain in the trial and be part of the final analysis set^11^. No formal stopping rules were defined.

We aimed to survey at least 30 intervention participants who accepted one of the offered intervention products. Therefore, we included all participants randomized to the intervention group until we reached these 30 completed interviews for this analysis.

### Pilot study data collection and outcomes

We used three different data sources: 1) the linked SHCS data for all characteristics; 2) the trial database that contained the participants’ product choice at baseline; and 3) a pilot study survey via phone call two to four weeks after baseline (Supplementary file Appendix 2).

The SHCS data collection is performed biannually by the treating physician^9^. All measurements apart from the laboratory measurements are self-reported. We defined alcohol use according to the AUDIT-C (Alcohol Use Disorder Identification Test Consumption) score^12^. We defined acceptance rate as the proportion of participants who accepted one of the three offered products and signed the respective intervention consent. In this pilot study survey, participants were asked whether they had used the products, about their experiences with the products, and their opinions on the flavor and nicotine concentration. Additionally, participants were queried about the usability of the products and whether they wished to switch to a different product or adjust the flavor or nicotine concentration.

### Pilot study statistical analysis

All analyses in the internal pilot study were descriptive. We used medians and interquartile ranges (IQR) for continuous variables and frequencies and percentages for categorical variables. For data management and analysis, we used R, version 4.5.1 (2025-0613; packages used are listed in Supplementary file Appendix 3).

## RESULTS

**Figure 1:**
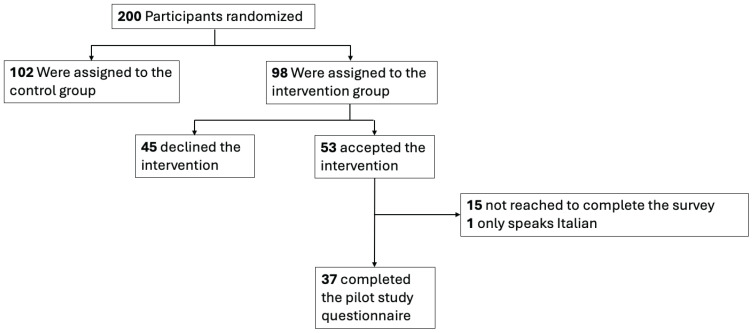
Flow-chart for the RETUNE internal pilot study. Participants were randomized between February and September 2025 in Switzerland (N=200)

Between 24 February and 3 September 2025, 200 participants were randomized (Figure 1); 98 participants were assigned to the intervention group, and 54.1% of those (53/98) accepted one of the offered nicotine substitute products. Thirty-seven of 53 (69.8%) intervention group participants completed the pilot study survey between 19 March and 2 October 2025.

The median age of intervention participants was 57 years (IQR: 43–64) (Table 1). Most participants were male, of European origin, and had no higher education degree. The median number of tobacco cigarettes smoked per day was 13 (IQR: 10–20), and the median number of years of smoking history was 16 (IQR: 10–25). Most participants were adherent to antiretroviral therapy, missing no more than one pill per month, had a viral load below 50 copies/mL, and a median CD4 count of 809 cells/mm^3^ (IQR: 590–1149). Substance use was common (63/98; 64.3%), mainly Cannabis (47/98; 48.0%), and injectable substance use was rare (3/98; 3.1%). More than one-third (38/98; 38.8%) had excessive alcohol use. There were no obvious numerical differences between participants in terms of acceptance rate, except for substantial variation across sites, ranging from 76.9% (center with the highest acceptance rate) to 9.1% (center with the lowest acceptance rate).

**Table 1:** Demographic and clinical characteristics of RETUNE pilot study participants (intervention arm only) randomized between February and September 2025 in Switzerland (N=98)

*Characteristics*	*Overall* *(N=98)* *n (%)*	*Intervention group acceptance* *(N=53)* *n (%)*	*Intervention group refusal* *(N=45)* *n (%)*
**Age** (years), median (IQR)	57 (43–64)	56 (41–63)	60 (46–65)
**Sex** (assigned at birth)			
Male	77 (78.6)	41 (77.4)	36 (80.0)
Female	21 (21.4)	12 (22.6)	9 (20.0)
**Higher education degree** ^a,b^	22 (22.4)	13 (24.5)	9 (20.0)
**Origin**			
Europe	85 (86.7)	47 (88.7)	38 (84.4)
Outside Europe	13 (13.3)	6 (11.3)	7 (15.6)
**Smoking status**			
Cigarettes per day, median (IQR)^c^	13 (10–20)	15 (10–20)	10 (7–20)
Years of tobacco smoking, median (IQR)^d^	16 (10–25)	17 (10–25)	15 (6–25)
**Center**			
1	40 (40.8)	27 (50.9)	13 (28.9)
2	13 (13.3)	10 (18.9)	3 (6.7)
3	2 (2.0)	0 (0)	2 (4.4)
4	12 (12.2)	9 (17.0)	3 (6.7)
5	9 (9.2)	5 (9.4)	4 (8.9)
6	22 (22.4)	2 (3.8)	20 (44.4)
**Missed antiretroviral therapy in the last 4 weeks**			
More than once	8 (8.2)	2 (3.8)	6 (13.3)
Once	12 (12.2)	7 (13.2)	5 (11.1)
Never	72 (73.5)	40 (75.5)	32 (71.1)
Not applicable	6 (6.1)	4 (7.5)	2 (4.4)
**Most likely mode of HIV acquisition**			
Men who have sex with men	45 (45.9)	25 (47.2)	20 (44.4)
Heterosexual contact	28 (28.6)	15 (28.3)	13 (28.9)
People who inject substances	13 (13.3)	9 (17.0)	4 (8.9)
Other/unknown	10 (10.2)	3 (5.7)	7 (15.6)
Perinatal	1 (1.0)	1 (1.9)	0 (0)
Blood products	1 (1.0)	0 (0)	1 (2.2)
**Viral load** (copies/mL)			
<50	92 (93.9)	49 (92.5)	43 (95.6)
50–399	3 (3.1)	2 (3.8)	1 (2.2)
≥400	3 (3.1)	2 (3.8)	1 (2.2)
**CD4 count** (cells/mm³), median (IQR)	809 (590–1149)	867 (526–1182)	763 (628–918)
**Diagnosis of depression** ^e,f^	8 (8.2)	7 (13.2)	1 (2.2)
**Substance use**			
Any substance use	63 (64.3)	34 (64.2)	29 (64.4)
Injectable substances^g^	3 (3.1)	2 (3.8)	1 (2.2)
Cannabis^h^	47 (48.0)	26 (49.1)	21 (46.7)
Other substances^i,j^	27 (27.6)	14 (26.4)	13 (28.9)
In a substance program	6 (6.1)	4 (7.5)	2 (4.4)
Excessive alcohol use^k^	38 (38.8)	22 (41.5)	16 (35.6)

a Higher education degree: defined as completed higher professional education or university degree above mandatory school (9 years in Switzerland) and finished apprenticeship.

b Missing (n=3).

c Missing (n=1).

d Missing (n=5).

e Assessed by a Swiss HIV Cohort Study physician or a psychiatrist.

f Missing (n=6).

g Frequency injectable substance use: weekly (n=1), monthly (n=1), less frequently (n=1).

h Frequency cannabis use: daily (n=18), weekly (n=10), monthly (n=8), less frequently (n=9), missing (n=2).

i Other substances: amphetamine/speed, cannabis, cannabidiol (CBD), cocaine, crack cocaine, crystal meth/tina, ecstasy, Gamma-hydroxybutyrate (GHB)/Gamma-butyrilactone (GBL), Ketamine, Lysergic acid.

j Frequency other substances: daily (n=3), weekly (n=3), monthly (n=7), less frequently (n=13), missing (n=1).

k Excessive alcohol consumption: defined as an AUDIT-C (Alcohol Use Disorder Identification Test Consumption) score ≥3 in females or ≥4 in males. IQR: interquartile range.

The most common reason for refusing all offered products was that the participants had no interest in quitting tobacco smoking (34/45; 75.6%) (Table 2). Some participants were not satisfied with the offered products (9/45; 20.0%), others were not willing to participate in a research project (5/45; 11.1%), and one participant (2.2%) was not willing to provide any contact information for supply coordination and therefore declined.

**Table 2:** Reported reasons of refusal of all the offered products among all participants randomized in the RETUNE intervention arm between February and September 2025 in Switzerland (N=45)

*Reasons for refusal ^a^*	*n (%)*
No interest in quitting tobacco smoking	34 (75.6)
Not satisfied by any of the offered products	9 (20.0)
Participant is not willing to participate in a research project	5 (11.1)
Concomitant cannabis use with tobacco	2 (4.4)
Participant is not willing to provide any contact information for supply coordination	1 (2.2)
Participant does not feel supported by any of the products	1 (2.2)
Participant will try quitting without the products and was already in smoking cessations consultation	1 (2.2)
Participant thinks nicotine products are harmful	1 (2.2)
Other^b^	2 (4.4)

a Multiple selection possible.

b Other priorities: too stressed because mother died 3 weeks ago (n=1), going to Asia for 3 weeks (n=1).

Almost two-thirds of the participants who accepted one of the offered interventions chose e-cigarettes (31/53; 58.5%), one-third chose nicotine patches (17/53, 32.1%), and four participants chose nicotine pouches (4/53; 7.5%) (Table 3). Among the 37 participants who completed the pilot study survey within four weeks after the start of the intervention, most reported that they continued their initially chosen product (27/37; 73.0%), some changed their product (10/37; 27.0%), and nobody stopped using the products (Table 4). The reported side effects were respiratory symptoms (6/28; 21.4%; not asked in patch users), skin reaction (6/37; 16.2%), nausea (3/37; 8.1%), headache (3/37; 8.1%), diarrhea (1/37; 2.7%), constipation (1/37; 2.7%), and mouth irritation (1/28; 3.6%; not asked in patch users); 54.1% (20/37) of the participants did not report any side effects. The side effects by product are provided in Supplementary file Appendix 4.

**Table 3:** Acceptance of one of the three offered products among all participants randomized in the RETUNE intervention arm between February and September 2025 in Switzerland (N=53)

*Offered intervention products*	*n/N (%)*
**E-cigarettes**	31/53 (58.5)
Menthol 3 mg/mL	0/31 (0)
Menthol 6 mg/mL	4/31 (12.9)
Menthol 16 mg/mL	3/31 (9.7)
Classic 3 mg/mL	4/31 (12.9)
Classic 6 mg/mL	11/31 (35.5)
Classic 16 mg/mL	9/31 (29.0)
**Nicotine patches**	17/53 (32.1)
Schema for strong smoker	9/17 (52.9)
Schema for medium/light smoker	8/17 (47.1)
**Nicotine pouches**	4/53 (7.5)
Cold 20 mg/g	3/4 (75.0)
Onyx 25 mg/g	1/4 (25.0)

**Table 4:** Results of pilot study survey conducted between March and October 2025 in Switzerland (N=37)

Results	n/N (%)
**Continued their product without changes during the first 4 weeks follow-up**	27/37 (73.0)
**Changed/modified product during the first 4 weeks follow-up**	10/37 (27.0)
E-cigarettes to nicotine patches	1/10 (10.0)
Nicotine pouches to nicotine patches	1/10 (10.0)
Changed liquid flavor	2/10 (20.0)
Decreased nicotine patch concentration	1/10 (10.0)
Decreased liquid nicotine concentration	2/10 (20.0)
Changed liquid flavor and decreased liquid nicotine concentration	2/10 (20.0)
Increased liquid nicotine concentration	1/10 (10.0)
Stopped the product entirely	0/37 (0)
Adverse event of special interest*	
Skin reaction	6/37 (16.2)
Respiratory symptoms (cough, phlegm, wheezing, sore throat)^#^	6/28 (21.4)
Nausea	3/37 (8.1)
Headache	3/37 (8.1)
Mouth irritation^#^	1/28 (3.6)
Diarrhea	1/37 (2.7)
Constipation	1/37 (2.7)
Emesis	0/37 (0)
Dizziness	0/37 (0)
Mouth ulcers^#^	0/28 (0)
Gingival pain^#^	0/28 (0)
Gingival bleeding^#^	0/28 (0)
No adverse events of special interest	20/37 (54.0)

* Several answer options per participant possible; stratified table by product provided in Supplementary file Appendix 4.

# Only asked among participants with e-cigarettes and nicotine pouches.

The experiences of 24 e-cigarette users are summarized in Supplementary file Appendix 5, where 65% (15/23) rated their experience overall as positive, and 35% (8/23) as neutral; 54% (13/24) of participants liked the flavor of the chosen liquids, while 33% (8/24) were neutral, and 26% (6/23) wished to change to another flavor when asked during the survey; 59% (13/22) of the participants rated the nicotine concentration of the liquids as ‘just right’, while 41% (9/22) rated the concentration as ‘too high’ or ‘too low’. Most participants perceived the e-cigarette as easy to handle (76%; 16/21) and useful to quit tobacco smoking (59%; 13/22). Among the 9 participants who used nicotine patches and filled in the survey, 75% (6/8) had an overall positive impression (Supplementary file Appendix 6); 56% (5/9) rated the nicotine concentration of the patches as ‘just right’, while 44% (4/9) rated the concentration as ‘too high’ or ‘too low’. All participants perceived the patches as easy to handle, and 75% (6/8) thought they were useful to quit tobacco smoking.

Among the four participants who chose nicotine pouches, two rated the flavor and the overall experience with the pouches as positive, and two as neutral (Supplementary file Appendix 7). No participant wanted another flavor, and all rated the nicotine concentration as ‘just right’. Two of the participants stated they had received enough information on the product, three believed that the pouches are useful to quit tobacco smoking, while one participant was neutral.

## DISCUSSION

This internal pilot study included the first 98 participants randomized to the intervention arm. The acceptance rate of one of the interventions was 54%, which is similar to our estimated 50%. E-cigarettes were the most popular product of the offered menu, followed by nicotine patches. In the subsample of surveyed intervention participants, all products (e-cigarettes, nicotine patches, nicotine pouches) were mostly rated positively. Most participants continued using the same product they had initially chosen (i.e. including the same flavor and nicotine strength) beyond the 4-week pilot phase, and no one stopped the cessation intervention altogether.

The proposed mechanism of action of the RETUNE trial is that participants will not only accept the offered products but also use them. This pilot study showed high engagement from the participants, which supports this proposed mechanism. Similarly, high engagement was observed by Carpenter et al.^13^ in a large smoking-cessation trial in the United States that offered e-cigarettes and also used an ‘opt-out’ approach, i.e. offering to participants irrespective of willingness to quit smoking. In a Swiss observational study, Auer et al.^14^ showed high uptake of smoking cessation counselling when offered to all smokers hospitalized with acute coronary syndrome in an ‘opt-out’ approach. The TwiCs design allowed us to implement such an opt-out approach in an efficient and fully embedded way in a running cohort study. Existing literature suggests that motivation to quit smoking is often overemphasized and may create barriers to accessing cessation support and participation in smoking cessation trials^15,16^. In comparison with a previous traditional ‘opt-in’ smoking cessation trial conducted in the SHCS^17^, our study achieved higher participation rates. However, direct attribution of this improvement to the trial design is challenging given the use of different interventions in the two trials. Notably, among the pilot study participants, smoking patterns were similar between participants who accepted and those who refused the intervention. This finding supports the concept that commonly used indicators, such as the number of cigarettes smoked per day, are unreliable predictors of an individual’s willingness to engage in smoking-cessation efforts^18,19^. Unsurprisingly, the most common reason for refusing all products was no interest in quitting tobacco smoking. Only two participants explicitly declined the offered intervention due to cannabis use, despite the common use of cannabis (48%) in the intervention group. If the RETUNE intervention also influences cannabis use or if ongoing cannabis use might interfere with the tobacco abstinence outcome, it remains to be investigated.

In trials using the TwiCs design, the acceptance rate of the offered intervention is a key aspect^7^. High refusal rates dilute the intention-to-treat estimate. Therefore, refusal should be anticipated and considered when calculating the trial sample size^20^. We calculated the sample size based on an anticipated acceptance of 50% and will continue recruitment after these reassuring results from the pilot study. Following the protocol, we will continuously reevaluate the acceptance rate. However, the pilot results show substantial differences in the acceptance rate across centers. These differences were unexpected, especially because there are no notable differences in participant characteristics, and the trial implementation process was comparable across centers. Further research is planned to investigate the reasons for varying acceptance rates across centers.

A further goal of the pilot study was to adapt the menu of offered products based on early participant feedback. E-cigarettes were the most popular product. However, only 60% of users were satisfied with the nicotine strength they had chosen. Therefore, we expanded the menu with two additional nicotine concentrations (0 mg/mL and 12 mg/mL). This change is reflected in the newest protocol version 1.2. Nicotine patches were the second most popular product and were also rated positively regarding handling and their perceived support for quitting tobacco smoking. The main issue was again nicotine dosing. Because patches are commercially available only in fixed strengths and are usually used in step-down regimens, we could not adjust the dosing of this product. Nicotine pouches were the least popular option. This may be explained by the average age of the study population, as nicotine pouches tend to be more common among younger people^21^. Moreover, participants without prior experience in using nicotine pouches may not have found them an appealing product.

To date, no randomized trial has offered e-cigarettes, nicotine patches, and nicotine pouches as a preference-based menu to tobacco smokers. Our findings on product popularity align with recent population-based data from England, where about 40% of smokers with a recent quit attempt used e-cigarettes, about 17% used over-the-counter nicotine replacement therapy including patches, and about 3% used nicotine pouches^22^. Results from a French pilot study among smokers with low socioeconomic status showed similar results for e-cigarettes and nicotine replacement therapy^23^.

### Limitations

Our study has the following limitations. First, we could not reach all participants within the first four weeks after randomization to complete the pilot study questionnaire (response rate: 70%). Therefore, it is possible that participants with negative experiences or generally lower motivation to provide data were underrepresented. This may have led to an overestimation of the positive perceptions of the intervention products and an underestimation of early discontinuation rates within the first four weeks. Second, the surveys were conducted by the study team via telephone, which may have influenced how participants responded to the questions, i.e. we cannot exclude social desirability bias. However, the real-time follow-up minimized recall-bias and helped to directly improve the implementation process. Third, the use of self-reported SHCS data could result in data misclassification, e.g. of smoking behavior, drug adherence, mode of HIV acquisition, depression, alcohol use, and substance use. Fourth, only four people chose the nicotine pouches which makes it difficult to assess experiences and side effects with this product. Fifth, the additional contact for the pilot study survey could potentially influence the intervention effectiveness. We plan to perform a corresponding sensitivity analysis in the effectiveness analysis to investigate this hypothesis.

## CONCLUSIONS

The results of this internal pilot support the feasibility of the RETUNE trial. The observed acceptance rate was similar to our estimated acceptance rate. E-cigarettes were the preferred product and satisfaction with all products was high. With minor changes to the intervention menu, we are continuing participant recruitment.

## Supplementary Material



## Data Availability

The data supporting this research are available from the following sources: the full trial protocol is available on the trial registry (NCT06789692), on the RETUNE website (retune-trial.com), and in the Supplementary file of this manuscript. The SHCS has a strict data sharing policy, but an anonymized analysis dataset is available upon request.

## References

[1] Weber MSR, Duran Ramirez JJ, Hentzien M, et al. Time trends in causes of death in people with hiv: insights from the swiss hiv cohort study. Clin Infect Dis. 2024;79(1):177-188. doi:10.1093/cid/ciae014 38214897 PMC11259222

[2] Trickey A, McGinnis K, Gill MJ, et al. Longitudinal trends in causes of death among adults with HIV on antiretroviral therapy in Europe and North America from 1996 to 2020: A collaboration of cohort studies. Lancet HIV. 2024;11(3):e176-e185. doi:10.1016/S2352-3018(23)00272-2 38280393 PMC11656032

[3] Johnston PI, Wright SW, Orr M, et al. Worldwide relative smoking prevalence among people living with and without HIV. AIDS. 2021;35(6):957-970. doi:10.1097/QAD.0000000000002815 33470609

[4] Rigotti NA, Kruse GR, Livingstone-Banks J, HartmannBoyce J. Treatment of tobacco smoking: A review. JAMA. 2022;327(6):566-577. doi:10.1001/jama.2022.0395 35133411

[5] Hartmann-Boyce J, Chepkin SC, Ye W, Bullen C, Lancaster T. Nicotine replacement therapy versus control for smoking cessation. Cochrane Database Syst Rev. 2018;5(5):CD000146. doi:10.1002/14651858.CD000146.pub5 29852054 PMC6353172

[6] Lindson N, Butler AR, McRobbie H, et al. Electronic cigarettes for smoking cessation. Cochrane Database Syst Rev. 2024;1(1):CD010216. doi:10.1002/14651858.CD010216.pub8 38189560 PMC10772980

[7] Amstutz A, Schönenberger CM, Speich B, et al. Characteristics, consent patterns, and challenges of randomized trials using the Trials within Cohorts (TwiCs) design - A scoping review. J Clin Epidemiol. 2024;174:111469. doi:10.1016/j.jclinepi.2024.111469 39032590

[8] Eldridge SM, Chan CL, Campbell MJ, et al. CONSORT 2010 statement: Extension to randomised pilot and feasibility trials. BMJ. 2016;355:i5239. doi:10.1136/bmj.i5239 27777223 PMC5076380

[9] Scherrer AU, Traytel A, Braun DL, et al. Cohort profile update: The Swiss HIV Cohort Study (SHCS). Int J Epidemiol. 2022;51(1):33-34. doi:10.1093/ije/dyab141 34363666

[10] Coart E, Bamps P, Quinaux E, et al. Minimization in randomized clinical trials. Stat Med. 2023;42(28):5285-5311. doi:10.1002/sim.9916 37867447

[11] Avery KN, Williamson PR, Gamble C, et al. Informing efficient randomised controlled trials: Exploration of challenges in developing progression criteria for internal pilot studies. BMJ Open. 2017;7(2):e013537. doi:10.1136/bmjopen-2016-013537 PMC5318608 28213598

[12] Bush K, Kivlahan DR, McDonell MB, Fihn SD, Bradley KA. The AUDIT alcohol consumption questions (AUDIT-C): An effective brief screening test for problem drinking. Ambulatory Care Quality Improvement Project (ACQUIP). Alcohol Use Disorders Identification Test. Arch Intern Med. 1998;158(16):1789-1795. doi:10.1001/archinte.158.16.1789 9738608

[13] Carpenter MJ, Wahlquist AE, Dahne J, et al. Effect of unguided e-cigarette provision on uptake, use, and smoking cessation among adults who smoke in the USA: A naturalistic, randomised, controlled clinical trial. EClinicalMedicine. 2023;63:102142. doi:10.1016/j.eclinm.2023.102142 37753443 PMC10518503

[14] Auer R, Gencer B, Tango R, et al. Uptake and efficacy of a systematic intensive smoking cessation intervention using motivational interviewing for smokers hospitalised for an acute coronary syndrome: A multicentre before-after study with parallel group comparisons. BMJ Open. 2016;6(9):e011520. doi:10.1136/bmjopen-2016-011520 PMC5051401 27650761

[15] Aveyard P, Begh R, Parsons A, West R. Brief opportunistic smoking cessation interventions: A systematic review and meta-analysis to compare advice to quit and offer of assistance. Addiction. 2012;107(6):1066-1073. doi:10.1111/j.1360-0443.2011.03770.x 22175545

[16] Danan ER, Joseph AM, Sherman SE, et al. Does motivation matter? Analysis of a randomized trial of proactive outreach to VA Smokers. J Gen Intern Med. 2016;31(8):878-887. doi:10.1007/s11606-016-3687-1 27071399 PMC4945562

[17] Gryaznov D, Chammartin F, Stoeckle M, et al. Smartphone app and carbon monoxide self-monitoring support for smoking cessation: a randomized controlled trial nested into the Swiss HIV Cohort Study. J Acquir Immune Defic Syndr. 2020;85(1):8-11. doi:10.1097/QAI.0000000000002396 32427723

[18] Schäfer J, Young J, Bernasconi E, et al. Predicting smoking cessation and its relapse in HIV-infected patients: The Swiss HIV Cohort Study. HIV Med. 2015;16(1):3-14. doi:10.1111/hiv.12165 24809704

[19] Oliver JA, Pacek LR, Locey EN, Fish LM, Hendricks PS, Pollak KI. Lack of utility of cigarettes per day cutoffs for clinical and laboratory smoking research. Addict Behav. 2019;98:106066. doi:10.1016/j.addbeh.2019.106066 31386967 PMC6708747

[20] Reeves D, Howells K, Sidaway M, et al. The cohort multiple randomized controlled trial design was found to be highly susceptible to low statistical power and internal validity biases. J Clin Epidemiol. 2018;95:111-119. doi:10.1016/j.jclinepi.2017.12.008 29277558 PMC5844670

[21] Brose L, Bunce L, Cheeseman H. Prevalence of nicotine pouch use among youth and adults in Great Britain-analysis of cross-sectional, nationally representative surveys. Nicotine Tob Res. 2026;28(2):213-222. doi:10.1093/ntr/ntae295 39788149 PMC12824946

[22] Jackson SE, Brown J, Buss V, Shahab L. Prevalence of popular smoking cessation aids in england and associations with quit success. JAMA Netw Open. 2025;8(1):e2454962. doi:10.1001/jamanetworkopen.2024.54962 39821398 PMC11742533

[23] Héron M, Le Faou AL, Ibanez G, Métadieu B, Melchior M, El-Khoury Lesueur F. Smoking cessation using preference-based tools: A mixed method pilot study of a novel intervention among smokers with low socioeconomic position. Addict Sci Clin Pract. 2021;16(1):43. doi:10.1186/s13722-021-00254-6 34193288 PMC8243481

