# Supplementary material for: Acceptance and preferences for different nicotine substitute products to reduce tobacco smoking in people living with HIV: Results from an internal pilot study of a randomized trial
Source: Tob Induc Dis. 2026 May 26;24:10.18332/tid/218987. doi: 10.18332/tid/218987 (PMC13205642; doi:10.18332/tid/218987)
Supplement: Supplementary file 1 [file TID-24-68-s1.pdf]

Study Protocol

Reduce tobacco use in people living with HIV in Switzerland: A pragmatic randomized trial within the Swiss HIV Cohort Study (RETUNE)

Study Type:	Other Clinical Trial according to ClinO, Chapter 4
Risk Categorisation:	Risk category A according to ClinO, Art. 61
Study Registration:	NCT06789692
Sponsor Investigator:	[redacted]<br>Division of Clinical Epidemiology,<br>University Hospital Basel<br>Totengässlein 3, 4051 Basel<br>+41 61 265 38 15<br>[redacted]
Investigated Intervention:	Offer of a menu of different tobacco smoking substitution products (nicotine pouches, electronic nicotine delivery systems, and nicotine patches)
Protocol ID	2024-02417
Version and Date:	Version 1.2 (dated 08.08.2025)

CONFIDENTIALITY STATEMENT

The information contained in this document is confidential and the property of the sponsor. The information may not - in full or in part - be transmitted, reproduced, published, or disclosed to others than the applicable Competent Ethics Committee(s) and Regulatory Authority(ies) without prior written authorisation from the sponsor except to the extent necessary to obtain informed consent from those who will participate in the study.

LIST OF PROJECT LEADERS AND OTHER KEY PERSONS INVOLVED IN THE TRIAL

Title	Names	Institution	Position	Role and Function/Responsibility in the project
[redacted]				

[redacted]

[redacted]

PROTOCOL SIGNATURE FORM

Study Title	Reduce tobacco use in people living with HIV in Switzerland: A pragmatic randomized trial within the Swiss HIV Cohort Study
Study ID	NCT06789692

The Sponsor-investigator, the other principal investigators, and all site-investigators have approved the protocol version 1.2 (dated 08/08/2025) and confirm hereby to conduct the study according to the protocol, current version of the World Medical Association Declaration of Helsinki, and ICH-GCP guidelines as well as the local legally applicable requirements.

Principal investigators:

Name: [redacted]

Date: ________________________ Signature: __________________________

Name: [redacted]

Date: ________________________ Signature: __________________________

Name: [redacted]

Date: ________________________ Signature: __________________________

Statistician:

Name: [redacted]

Date: ________________________ Signature: __________________________

Local Investigator at study site:

Site: University Hospital Basel

Local Investigator: [redacted]

Date: ________________________ Signature: __________________________

Site: University Hospital Zurich

Local Investigator: [redacted]

Date: ________________________ Signature: __________________________

Site: Inselspital Bern

Local Investigator: [redacted]

Date: ________________________ Signature: __________________________

Site: Cantonal Hospital St. Gallen

Local Investigator: [redacted]

Date: ________________________ Signature: __________________________

Site: University Hospital Lausanne

Local Investigator: [redacted]

Date: ________________________ Signature: __________________________

Site: University Hospital Geneva

Local Investigator: [redacted]

Date: ________________________ Signature: __________________________

Site: Cantonal Hospital Aarau

Local Investigator: [redacted]

Date: ________________________ Signature: __________________________

TABLE OF CONTENTS

Table of Contents **7**

1 STUDY SYNOPSIS **10**

2 BACKGROUND and rationale **14**

2.1 People living with HIV in Switzerland – addressing the shift of mortality and morbidity *14*

2.2 Tackling a major health burden among PLWH *14*

2.3 Electronic nicotine delivery systems *15*

2.4 Nicotine pouches *16*

2.5 Nicotine patches *17*

2.6 New ways to include more smokers in smoking cessation programs *17*

2.7 Using an innovative design to generate randomized evidence in cohort studies *18*

2.8 Inequality in smoking cessation research regarding low socioeconomic status and HIV *18*

2.9 Scientific impact *18*

3 Study OBJECTIVES and Design **19**

3.1 Hypothesis and objectives *19*

3.2 Primary and secondary endpoints *21*

3.3 Study design *22*
3.3.1 Allocation concealment and blinding 23
3.3.2 Randomization 23

4 Study POPULATION and Study procedures **23**

4.1 Inclusion and exclusion criteria, justification of study population *23*
4.1.1 Inclusion criteria 23
4.1.2 Exclusion criteria 23
4.1.3 Justification of the study population 24

4.2 Recruitment, screening, and informed consent procedure *24*
4.2.1 Screening and recruitment 24
4.2.2 Informed consent 24

4.3 Study intervention *25*

4.3.1 Description of electronic nicotine delivery system (e-cigarettes) *25*
4.3.2 Description of nicotine pouches 25
4.3.3 Description of nicotine patches 25
4.3.4 Comparator 25

4.4 Study procedures *26*
4.4.1 General Setting 26
4.4.2 Trial procedures and data collection 26
4.4.3 Storage conditions and supply of intervention products 29

4.5 Withdrawal and discontinuation *29*

4.6 Compliance with the intervention *29*

4.6.1 Compensation for participants 30
4.6.2. Compensation for participating trial centers 30

4.7 Participation in other clinical trials 30

4.8 Patient and public involvement and the participant advisory board 30

5 STATISTICS AND METHODOLOGY 30

5.1 Sample size calculation 30

5.2 Adaptive readjustment 31

5.3 Internal pilot study 32

5.4 Description of statistical methods 33
5.4.1 Dataset to be analyzed 33
5.4.2 Primary analysis 33
5.4.3 Secondary analyses 33
5.4.4 Interim analysis 37
5.4.5 Subgroup analysis 37

5.5 Handling of missing data and drop-outs 37

6 Regulatory Aspects AND SAFETY 38

6.1 Local regulations / Declaration of Helsinki 38

6.2 (Serious) Adverse Events: Definitions, collection, and assessment 38
6.2.1 Adverse Events (AE) 38
6.2.2 Adverse Events of Special Interest (AESI) 38
6.2.3 Serious Adverse Event (SAE) 38
6.2.4 Collection of AE, SAE, AESI 39
6.2.5 Assessment of causality and severity 39
6.2.6 Reporting of SAEs (see ClinO, Art. 63)[97] 39

6.3 (Periodic) safety reporting and reporting of general progress 39

6.4 Pregnancy 40

6.5 Amendments 40

6.6 Notification and reporting upon completion, discontinuation or interruption of the study 40

6.7 Insurance 40

7 FURTHER Aspects 41

7.1 Overall ethical considerations 41

7.2 Risk-benefit assessment 41

7.3 Time plan and trial duration 42

7.4 Process evaluation 42

8 Quality CONTROL AND Data protection 42

8.1 Quality measures 42

8.2 Data recording and source data 43

8.3 Confidentiality and coding 43

8.4 Retention and destruction of study data and biological material *45*

9 Monitoring and Registration **45**

10. Funding / Publication / declaration of Interest **45**

11. REFERENCES **47**

GLOSSARY OF ABBREVIATIONS

AE	*Adverse Event*
AESI	*Adverse Event of Special Interest*
BASEC	*Business Administration System for Ethical Committees*
CACE	*Complier Average Causal Effect*
CVD	*Cardiovascular Disease*
DAG	*Directed Acyclic Graph*
e-cigarettes	*electronic cigarettes*
eCRF	*electronic Case Report Form*
EKNZ	*Ethic committee of Northern and Central Switzerland*
FOPH	*Federal Office of Public Health*
FSVA	*Federal Food Safety and Veterinary Office*
HDL	*High Density Lipoprotein*
ITT	*Intention-To-Treat*
LDL	*Low Density Lipoprotein*
PLWH	*People Living With HIV*
PPI	*Patient and Public Involvement*
RCT	*Randomized Clinical Trial*
SAE	*Serious Adverse Events*
SHCS	*Swiss HIV Cohort Study*
TwiCs	*Trial within Cohorts*

1 STUDY SYNOPSIS

**Sponsor / Sponsor-Investigator**	Division of Clinical Epidemiology, University Hospital Basel<br>Totengässlein 3, 4051 Basel<br>Represented by: Prof. Matthias Briel, MD PhD<br>+ 4161 61 265 38 15<br>Matthias.Briel@usb.ch
**Study Title**	Reduce tobacco use in people living with HIV in Switzerland: A pragmatic randomized trial within the Swiss HIV Cohort Study
**Short Title / Study ID**	RETUNE
**Protocol Version and Date**	Version 1.2 (dated 08.08.2024)
**Study Registration**	NCT06789692
**Study Category and Rationale**	Risk category A, Other clinical trials<br>Nicotine pouches and electronic cigarettes (electronic nicotine delivery systems and liquids) are regulated by the law on alimentary goods in Switzerland. We therefore follow the rules of the Federal Office of Public Health (FOPH) and the Federal Food Safety and Veterinary Office (FSVA) for the import and use of nicotine-containing e-liquids. The risk is considered to be low.<br>Nicotine patches are an authorized, established, low risk medical product.
**Background and Rationale**	Due to highly effective antiretroviral therapy, people living with HIV (PLWH) in Switzerland have a close-to-normal life expectancy. Among PLWH, there has been a shift of focus from HIV-related health issues to non-communicable diseases, especially cardiovascular diseases (CVDs) and cancer. Smoking, a prominent risk factor for both diseases, takes a key role in view of the high number of smokers among PLWH.<br>New approaches to smoking cessation focus on harm reduction by substituting tobacco cigarettes by less harmful alternatives. Conventional nicotine replacement therapy like nicotine patches is well established and effective against withdrawal symptoms after quitting. However, these products are exceptionally expensive and the missing “nicotine hit” often limits therapy adherence. The long-term abstinence rate remains low. Electronic cigarettes (e-cigarettes) and nicotine pouches as alternative nicotine replacement therapies have the potential to overcome these problems and are accessible.<br>E-cigarettes play a growing role in smoking cessation therapy. Evidence from randomized trials shows superiority over conventional nicotine replacement therapies, which is probably based on the better imitation of the smoking experience. Although e-cigarettes are not without concerns, there is consensus that they are significantly less harmful than tobacco cigarettes.<br>Tobacco-free nicotine pouches, delivering nicotine through oral mucosa, are a relatively novel option for nicotine substitution. Similar to e-cigarettes, nicotine pouches are relatively affordable and can address the limitation of poor imitation of the “nicotine hit” seen with traditional nicotine replacement therapy. To the best of our knowledge, no trials have investigated the potential of nicotine pouches for smoking cessation so far.<br>With this trial, we also want to address frequent shortcomings of smoking cessation trials, such as restrictive inclusion criteria and highly controlled interventions, by

	offering a menu of different nicotine substitute products (e-cigarettes or nicotine pouches or nicotine patches) and by using a novel pragmatic trial design, the Trials within Cohorts (TwiCs) design. We plan to recruit tobacco smokers in the Swiss HIV Cohort Study (SHCS), regardless of their willingness to quit. This so called "opt-out-approach" has been suggested as a new promising approach for smoking cessation trials to increase generalizability of the results. The TwiCs design optimally allows to implement and evaluate the "opt-out" approach embedded in the SHCS.
**Risk / Benefit Assessment**	This trial investigates a low-risk intervention in a pragmatic way. The risk for the trial participants will be low and the application of the different products reflect real-life practice.<br>All interventions used in this trial (e-cigarettes, nicotine pouches and nicotine patches) are approved by the Swiss authorities and freely available on the market. The nicotine patches are a drug and approved by Swissmedic.<br>All products may cause local reactions and irritation of the skin or mucous membrane. Accordingly, the trial participants will be informed to discontinue the products and to contact their treating physician in case of corresponding side effects. Adverse events will be collected using surveys and routinely collected data from the SHCS.<br>The intervention is designed to help trial participants quit smoking tobacco cigarettes. Given the major health burden caused by tobacco smoking, especially in PLWH, the potential benefit of the trial intervention clearly outweighs the potential low risks. If proven effective, our intervention can be translated directly into clinical practice (in PLWH and possibly other settings) to reduce the burden of tobacco smoking in terms of morbidity and mortality.
**Objective(s)**	**Primary objective:** The purpose of this trial is to test the effectiveness of offering a menu of different nicotine substitute products (e-cigarettes, nicotine pouches, nicotine patches in addition to usual of care) for tobacco smokers in the SHCS in achieving self-reported 7-day tobacco cigarette abstinence at 6 months.<br><br>**Secondary objectives:**<br>● To assess the effect of offering the preference-based smoking cessation intervention on tobacco smoking cessation (defined as 7-day tobacco cigarette abstinence) at 12 and 24 months.<br>● To assess the effect of offering the preference-based smoking cessation intervention on (i) reduction of smoked tobacco cigarettes per day; (ii) reduction of cardiovascular risk and (iii) occurrence of cardiovascular events or death.
**Endpoint(s)**	**Primary endpoint**<br>Tobacco smoking status (yes/no) measured as self-reported abstinence in the last 7 days at 6-month visit (window: 120-270 days)<br>**Secondary endpoints**<br>1. Tobacco smoking status (yes/no) measured as self-reported abstinence in the last 7 days at 12-month visit (window: 271-450 days) and 24-month visit (window: 630-810 days).<br>2. Mean change in the self-reported number of tobacco-based cigarettes smoked per day at 6,12, and 24 months.<br>3. Self-reported use of any nicotine containing product other than tobacco cigarettes (yes/no). If yes, self-reported use of e-cigarettes (yes/no) or nicotine pouches (yes/no) or patches (yes/no) or other (yes/no) after 6

	(window: 120-270 days), 12 (window: 271-450 days) and 24 (window: 630-810 days) months.<br>4. Self-reported abstinence of any nicotine containing product (yes/no) after 12 (window: 271-450 days) and 24 (window: 630-810 days) months.<br>5. Mean change in High Density Lipoprotein (HDL), Low Density Lipoprotein (LDL), and total Cholesterol (mmol/l) at 6,12, and 24 months.<br>6. Mean change in systolic and diastolic blood pressure (mmHg) at 6,12, and 24 months.<br>7. Mean change in body weight (kg) from baseline to 6,12 and 24 months.<br>8. Mean change in SCORE2 score at 6, 12, and 24 months.<br>9. Occurrence of cardiovascular events (myocardial infarction, coronary angioplasty/stenting, coronary artery by-pass grafting, carotid endarterectomy, stroke, deep vein thrombosis, pulmonary embolism, heart transplantation) at 6,12, and 24 months.
**Study Design**	Randomized clinical trial according to the "Trials within Cohorts" (TwiCs) design approach. Individual 1:1 randomization, stratified by minimization variables (see below).
**Statistical Considerations**	The primary analysis will use the intention-to-treat set and a logistic regression model adjusted for the minimization variables (region (French vs. German speaking part of Switzerland), men having sex with man (yes/no), current drug user (yes/no), and the number of cigarettes per day) to test for superiority of the intervention (vs control) at a significance alpha level of 0.05.<br>In secondary analyses, we will assess per protocol estimates for the primary endpoint using observational causal inference methods. No interim analysis is planned.
**Inclusion- / Exclusion Criteria**	**Inclusion criteria:** SHCS participants aged 18 or older, living with HIV, accessing routine HIV care at one of the involved trial sites, who smoke one or more tobacco-based cigarettes per day at the day of trial inclusion and signed the randomization consent.<br>**Exclusion criteria:** SHCS participants who are, besides tobacco cigarettes, already using e-cigarettes or nicotine pouches or nicotine patches. Pregnant women are excluded.
**Number of Participants with Rationale**	We assume a smoking cessation rate of 8.5% in the control arm (based on SHCS data), a 20% cessation rate in the intervention arm (based on external evidence of similar trials), and an attrition rate of 3% (based on SHCS data). Considering a low uptake of only 50% in the intervention and thus dilution of the intervention effect, we aim at enrolling a total 972 participants to achieve 80% power for a two-sided alpha level at 5%. We will update our sample size during recruitment depending on observed uptake rates, monitored at predefined recruitment timepoints.
**Study Intervention**	Offer of menu of different nicotine substitute products, consisting of e-cigarettes or nicotine pouches or nicotine patches in addition to standard of care. This menu will be offered by the treating physician during the routine cohort visit and the participant can choose one product to test as an alternative to tobacco smoking. The products will be handed out directly after the consultation and are provided free of charge for 6 months.
**Control Intervention**	Standard smoking cessation counselling according to routine care in the SHCS. The treating physician is allowed to prescribe medication or further smoking cessation counselling, if indicated or wished by the participant. However, participants in the control group will not be offered the intervention menu and no recommendations to use e-cigarettes or nicotine pouches will be made.

**Study procedures**	The trial endpoints will be collected during regular cohort visits, which take place every 6 months.<br>The following parameters will be collected (part of routine care): smoking status, number of cigarettes smoked in the last 7 days, use of nicotine patches, nicotine chewing gums, snus, nicotine pouches, pipe, snuff, IQOS, and e-cigarettes. In addition, information on systolic and diastolic blood pressure (mmHg), body weight (kg), HDL-, LDL-, and total Cholesterol (mmol/l), hospitalizations and death are routinely collected during SHCS visits and used for trial purposes.<br>Additional safety endpoints and adherence information in the intervention group are collected retrospectively (not part of the SHCS routine data collection).
**Study Duration and Schedule**	3 years<br>First patient in: February 2025<br>Last patient out: December 2027
**Sponsor Investigator**	[redacted]<br>Deputy Head of Division<br>Division of Clinical Epidemiology<br>University Hospital Basel<br>Totengaesslein 3, 4051 Basel, Switzerland<br>+ 4161 265 3815<br>[redacted]
**Study Center(s)**	In this multicenter trial 7 institutions will be involved:<br>**University Hospital Basel** (Klinik für Infektiologie und Spitalhygiene, Petersgraben 4, 4031 Basel and Division of Clinical Epidemiology, Totengässlein 3, 4051 Basel)<br>**University Hospital Zurich** (Klinik für Infektionskrankheiten und Spitalhygiene, Rämistrasse 100, 8091 Zürich)<br>**Inselspital Bern** (Universitätsklinik für Infektiologie, Freiburgstrasse 16, 3010 Bern)<br>**Cantonal Hospital St. Gallen** (Klinik für Infektiologie und Spitalhygiene, Rorschacherstrasse 95, 9007 St. Gallen)<br>**University Hospital Lausanne** (Service des maladies infectieuses, Rue de Bugnon 46, 1011 Lausanne)<br>**University Hospital Geneva** (Service des maladies infectieuses, Rue Gabrielle-Perret-Gentil 4, 1205 Genève)<br>**Cantonal Hospital Aarau** (Klinik für Infektiologie und Infektionsprävention, Haus 7, Tellstrasse 25, 5001 Aarau)
**Data privacy**	The data of the participants will be kept encrypted. Identifying patient data will only be visible for the principal investigators and for staff at the respective SHCS sites. Coding keys will be kept at the treating SHCS site, according to the SHCS protocol.<br>No blood sampling will be collected as part of this study.
**Ethical consideration**	This project will be conducted in accordance with the research plan outlined in this protocol and with principles according to the current version of the Declaration of Helsinki as well as all national legal requirements and guidelines as applicable.<br>This protocol will be reviewed by the Ethikkommission Nordwest- und Zentralschweiz (EKNZ, Ethics Committee of Northern and Central Switzerland) as the leading ethics committee before the start of the trial.<br>All participants in the SHCS and in the RETUNE trial are participating voluntarily. All intervention products can be declined and discontinued, and participants will continue receiving best available care as part of the SHCS. It is possible to not

	participate in the RETUNE trial but be a participant in the SHCS. The treating study centers, who have many years of experience, ensure the best possible care for the trial participants.<br>The nicotine substitute products may have potential risks and participants will be informed about these. Only products that are approved and freely available will be used. However, the health benefit of reducing tobacco smoking outweighs potential side-effects from the harm reduction products.<br>The evidence generated by this trial will help improve national and international guidelines with respect to smoking cessation for PLWH.
**GCP Statement**	This study will be conducted in compliance with the protocol, the current version of the Declaration of Helsinki, the ICH-GCP, the HRA as well as other locally relevant legal and regulatory requirements.

2 BACKGROUND AND RATIONALE

2.1 People living with HIV in Switzerland – addressing the shift of mortality and morbidity

People living with HIV (PLWH) have a close-to-normal life expectancy due to highly effective antiretroviral therapy that has been rolled out over the last decades.[1] Among PLWH, there has been a shift of focus from HIV-related health issues to non-communicable diseases, especially cardiovascular diseases (CVDs)[1-4] and cancer.[5,6] By 2030, an estimated 84% of PLWH will suffer from a non-communicable disease, and 78% will have a CVD.[7] Two meta-analyses, including predominantly PLWH from Europe and North America, conclude a doubled overall risk for CVDs in this population compared to the general population.[2,8,9] Moreover, the incidence of lung cancer, which is heavily driven by tobacco smoking, is higher in PLWH.[10,11] While HIV-related morbidity and mortality have significantly decreased, non-communicable diseases and their risk factors represent the most documented health burden among PLWH, especially in high-income countries such as Switzerland.[12]

The Swiss HIV Cohort Study (SHCS) (Business Administration System of Ethic Committees (BASEC) number 2023-02080) established in 1988, is a multicenter, prospective, observational, nationwide cohort study.[13] PLWH are recruited at all university hospitals in the country and at various other clinics and practices. The cohort included about 9’500 active participants.[14]

2.2 Tackling a major health burden among PLWH

Smoking is one of the leading causes of death and is responsible for approximately 7.7 million deaths per year worldwide.[15] Tobacco smoking is also a key contributor for CVD and thus overall a major health burden among PLWH.[5,16] In 2017, 34% of participants in the SHCS smoked 3 or more cigarettes per day.[17] Today, smokers with HIV lose more life years to smoking than to HIV.[5] There is widespread consensus that nicotine, while addictive, is not responsible for smoking-associated diseases such as CVD and cancer.[18,19] Therefore, harm reduction interventions using nicotine substitute products have become a mainstay of tobacco smoking cessation programs. Conventional nicotine replacement therapy, such as patches or gums, are well established and effective against withdrawal symptoms after quitting, but these products are exceptionally expensive in Switzerland and are highly regulated.[20] Furthermore, the missing “nicotine hit” due to the slow release of nicotine often limits therapy adherence. Electronic cigarettes (e-cigarettes) and nicotine pouches as alternative nicotine substitute products have the potential to overcome

some of these problems and are already in widespread but unregulated use. Their effectiveness needs to be assessed in high-quality, randomized, large-scale, clinical trials.

2.3 Electronic nicotine delivery systems

E-cigarettes, also referred as vapors or electronic nicotine delivery systems, are devices imitating tobacco cigarettes in their use and properties. E-cigarettes heat up nicotine containing liquids to inhale nicotine in the form of aerosols (vapor) in contrast to smoke like in traditional tobacco cigarettes. The liquids are usually available in different flavors and components. By avoiding burning tobacco, e-cigarettes produce a significantly lower amount of harmful chemicals than tobacco cigarettes.[21] Despite potential risks of e-cigarettes, there is widespread agreement that replacing traditional tobacco cigarettes with e-cigarettes can significantly reduce exposure to toxins and carcinogens.[22]

Furthermore, in Switzerland e-cigarettes are considered to be alimentary goods and fall under the "*Lebensmittel- und Gebrauchsgegenständeverordnung*" (LGV SR 817.02). Currently, e-cigarettes are not classified as medicine or medical devices in any country in the world.[21] The resulting competitive market keeps the prices of e-cigarettes low, especially when compared with traditional nicotine replacement therapies like nicotine patches, gums, sprays, lozenges or inhalers. E-cigarettes should be understood as a heterogeneous group of devices across different generations with different characteristics, such as different capacities in delivering nicotine. Newer generations of e-cigarettes proved to be more effective in delivering nicotine.[23]

While nicotine is mainly responsible for the neuropharmacological dependence on tobacco smoking, bio-behavioral factors and smoking related stimuli also play important roles in smoking cessation.[24] This seems an obvious reason for the mediocre success of conventional nicotine replacement products, which substitute nicotine but poorly mimic the smoking experience. E-cigarettes come closer to the smoking routine and mimic experiences of smoking, such as the feeling of holding a cigarette, or inhaling smoke into the throat ("throat hit") or exhaling the cloud of smoke.[25] Moreover, nicotine is absorbed with e-cigarettes at a similar fast rate as with conventional tobacco cigarettes, which enables the "nicotine hit" that plays an important role for an addictive behavior. By meeting the needs of smokers better than conventional nicotine replacement therapies, e-cigarettes also carry the potential to act as a long-term substitute.[26]

Although there is still a lack of empirical data (especially long-term), the current evidence suggests that e-cigarettes are notably less harmful than conventional cigarettes with respect to the development of cancer and lung disease.[27] A danger that should not be underestimated is the strong addiction potential of e-cigarettes, which may tempt people to smoke and needs to be tackled in the smoking prevention programs among adolescents. This makes the correct use of e-cigarettes in smoking prevention even more important.

A Cochrane review from 2024, which included 88 studies, 47 of which were randomized clinical trials (RCT), showed that e-cigarettes improved cessation rates compared with conventional nicotine replacement therapy and did not lead to more adverse events.[28] While e-cigarette use is still controversial among health professionals and policy makers, the evidence is pointing in direction of promoting e-cigarettes as a reasonable smoking cessation tool to save lives from smoking. For these reasons, the accessible and low-cost e-cigarettes represent an important pillar for our smoking cessation intervention.

Figure 1: E-cigarette (Endura T20-S-Kit)

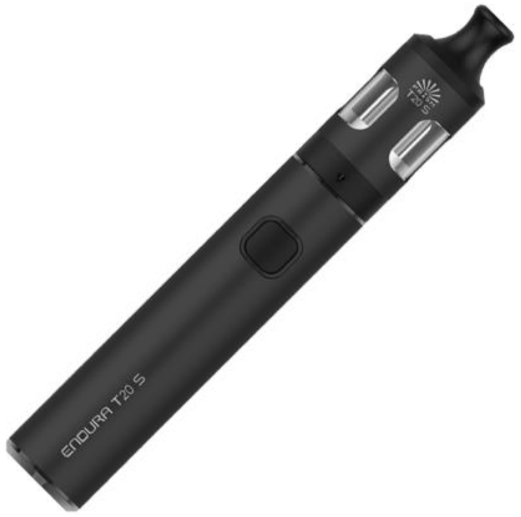

2.4 Nicotine pouches

Tobacco-free nicotine pouches are nicotine products for oral use. They consist of a small pouch, which is filled with a nicotine-containing matrix. The nicotine comes either from tobacco plants or is produced synthetically. In contrast to the widespread used snus in Scandinavia, nicotine pouches do not contain tobacco. In the application, however, they do not differ, the pouches are clamped between the lip and the palate and provide the nicotine through the oral mucosa. Similarly to e-cigarettes, nicotine pouches are available in different flavors.

Following the concept of harm reduction, the use of nicotine pouches has grown as a tool for nicotine substitution. Nicotine pouches offer several advantages over conventional nicotine replacement therapy. Nicotine pouches are comparatively affordable, both in comparison with tobacco cigarettes and with nicotine replacement therapy and they are convenient in use. According to a 2022 study by Jakub et al., nicotine pouches are the cheapest form of nicotine delivery in Switzerland.[29]

Regarding the safety of nicotine pouches, it is difficult to draw sound conclusions due to the small number of studies. However, the available evidence from pharmacokinetic studies suggests that nicotine pouches were safe and well tolerated in healthy participants. No serious adverse events were recorded.[30,31] In 2013, McNeill et al. introduced the concept of the risk continuum for various nicotine products. Classical tobacco cigarettes and other forms of inhaled tobacco (combustible tobacco products) represent the most dangerous form. Oral tobacco products (non-combustible tobacco products) represent an intermediate stage and non-combustible nicotine products like traditional nicotine replacement therapies represent the least dangerous stage.[32] A study based on nicotine pouches chemical analysis of the most common toxicants found in smokeless tobacco products suggests that nicotine pouches should be positioned similarly to nicotine replacement therapies in this continuum.[33] Further, an in vitro study assessing the influence of nicotine pouches on gingiva organotypic cultures classified them as non-mutagenetic, non-genotoxic and non-cytotoxic compared to conventional cigarettes.[34] A described problem of nicotine pouches are occasionally high nicotine concentrations, which have a large dependence potential.[35] Currently, no data are available on long-term effects.[36]

Despite these concerns, but especially because of the unanimously recognized harmfulness of tobacco cigarettes, nicotine pouches are an increasingly accepted tool for harm reduction in smokers. When substituting nicotine, it is important to consider at what rate and in what amounts nicotine is being absorbed. Conventional cigarette smoking is particularly effective in absorbing nicotine. A major reason why many smokers do not accept nicotine replacement therapy is that they deliver nicotine much slower than cigarettes.[37] Nicotine pouches overcome this weakness and deliver nicotine faster in higher doses to achieve satisfactory plasma nicotine concentrations.[30,31] To the best of our knowledge, so far no trials have investigated the potential of nicotine pouches for smoking cessation.

Figure 2: Nicotine pouches[33]

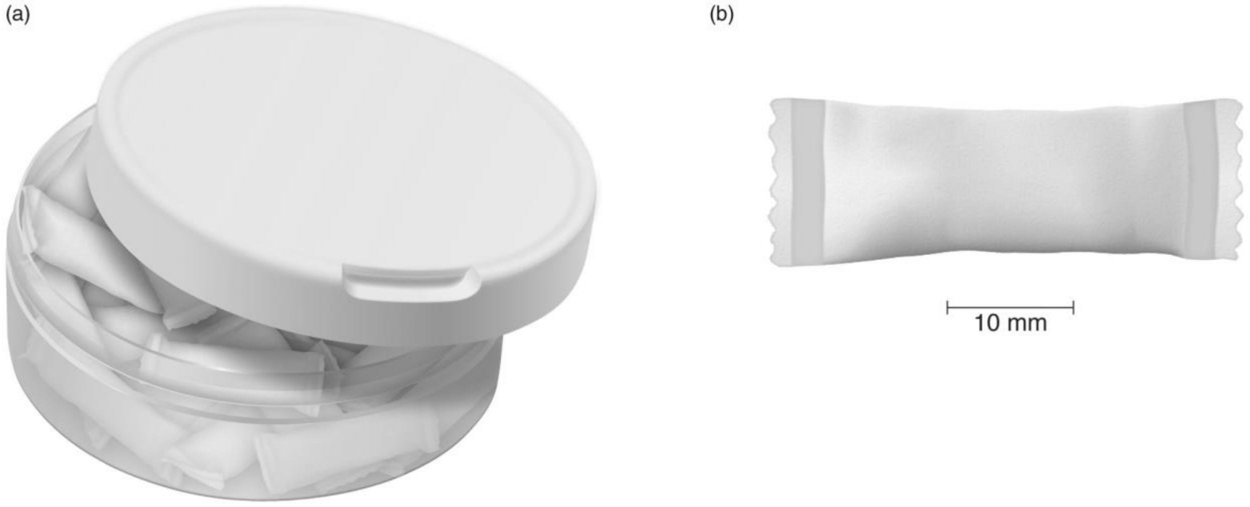

2.5 Nicotine patches

Nicotine-containing transdermal patches are a widely used form of nicotine replacement therapy and an established standard of care against withdrawn symptoms.[38] Due to the simple application, they achieve a high compliance rate. They are equally effective as other traditional nicotine replacement therapy (e.g. nicotine gums, nicotine inhalers) and more effective than placebo for smoking cessation.[39,40] The transdermal nicotine uptake is slow and nicotine patches need about one hour to reach the maximum nicotine concentration. Afterwards, they create a steady state for up to 24 hours. Therefore, nicotine patches do not imitate any of the pharmacodynamic and behavioral properties of smoking.[40] This may limit their effectiveness. The most common side effects are skin reactions.

2.6 New ways to include more smokers in smoking cessation programs

Smoking cessation trials often struggle with suboptimal recruitment and limited applicability of the results. A common problem is often the strict inclusion criteria, only allowing smokers willing to quit in a specific timeframe to participate in the trial. This selection leads to a lack of applicability of trial results in clinical practice and establishes a hurdle for smokers to receive smoking cessation support. This problem is present in many large completed and ongoing smoking cessation trials investigating the potential of e-cigarettes in nicotine substitution.[41-44]

One possible solution is the so called “opt-out” approach, in which all smokers are provided with smoking cessation aids and counseling, not only those who express a willingness to quit as in the

classic “opt-in” approach.[45-47] It has been shown that smokers who want to quit and smokers without this explicit intention have similar quit rates, provided they receive appropriate support.[48,49] Smoking cessation studies according to the "opt-out" design can be more easily translated into clinical practice. The goal of our trial is to provide evidence for a future large-scale public health intervention that may be offered to all smokers during a consultation. The “Trials within Cohorts” (TwiCs) design (see section 2.7) is the ideal trial design to implement and evaluate the “opt-out” approach embedded in a cohort study.

2.7 Using an innovative design to generate randomized evidence in cohort studies

RCTs represent the gold standard in terms of evidence generation on therapeutic or prophylactic interventions in clinical research.[50] However, RCTs are expensive and often challenging to plan and conduct and findings from RCTs using highly controlled procedures are often difficult to translate into routine care.[51] Therefore, more efficient and applicable trial designs are needed. TwiCs is a randomized trial design whereby participants are recruited within a prospective cohort study and give their consent not only to regular data collection at cohort visits, but at the same time, they are asked for their consent to be randomized into future pragmatic trials, evaluating low-risk interventions, nested in the cohort.[52,53] Participants are informed that they will be contacted in case they are randomized into the intervention arm and may accept or decline the intervention. In case they are randomized to the control arm (usual care), they will not be contacted but followed continuously in the frame of the cohort study. Participants in both arms are not aware of the other arm, which reduces disappointment effects, cross-over and withdrawals.[54] The multistep consent procedure in TwiCs mimics a usual care setting in which people are asked to consent to treatment options while not being told about treatments they cannot access. Published evidence suggests that TwiCs are efficient in terms of enhanced recruitment and completeness of follow-up, and provide better external validity due to the pragmatic approach of the trial.[55] The TwiCs design has been adopted by several cohorts across the world and in a variety of medical fields such as breast cancer, bone metastases, mental health, or care of elderly people – and recently in the SHCS.[56]

2.8 Inequality in smoking cessation research regarding low socioeconomic status and HIV

Despite consensus about the need for effective smoking cessation interventions, PLWH represent a neglected population with respect to smoking cessation studies.[57] Furthermore, it is known that people with low-socioeconomic status suffer more severely from the burden of smoking and encounter various barriers to smoking cessation.[58,59] Thus, there is still a lack of effective smoking cessation interventions for these people.[60] Data on the effectiveness of offering a preference-based smoking cessation intervention taking into account socioeconomic parameters are lacking, as is knowledge about disadvantaged groups within PLWH in relation to smoking cessation interventions. The SHCS with its representative population holds the opportunity to close some of these knowledge gaps.

2.9 Scientific impact

TwiCs is a pragmatic trial design with the goal to mimic a real-life situation and testing interventions that can be adopted into everyday medical practice. Tobacco smoking represents a major health burden in PLWH. Quitting tobacco smoking is the key to reduce mortality.[67] There is consensus among patient representatives, researchers, and clinicians that new and effective smoking cessation interventions are needed. Further, we will challenge the established pattern of

smoking cessation trials, which presupposes smokers' willingness to quit. Evidence from the literature shows that (1) the lengthy process of participating in a smoking cessation program (opt-in principle) discourages many smokers from quitting and that (2) smokers who have the intention to quit and those without this intention show similar quit rates with appropriate support. With this trial, we expect to achieve a doubling of smoking cessation rates compared to the control group. Additionally, we aim to generate needed evidence to better assess the effectiveness and safety of e-cigarettes and nicotine pouches.

With the implementation of the TwiCs for the first time in Switzerland, we hope to pave the way to improve randomized evidence generation within a large-scale national cohort study. The SHCS will thus play a pioneering role both nationally and internationally and increase its image of an innovative nationwide cohort study.

3 STUDY OBJECTIVES AND DESIGN

3.1 Hypothesis and objectives

Hypothesis: The offer of a preference-based smoking cessation intervention menu, consisting of e-cigarettes, nicotine pouches and nicotine patches, in addition to usual care is safe and superior to reduce tobacco smoking among PLWH in the SHCS after 6 months compared with usual care alone.

Primary estimand: The primary estimand is described in *Table 1*. Intercurrent events and their handling are described in *Table 2.* The description of the primary estimand follows current guidelines.[68,69]

Table 1: Definition of the primary estimand (primary analysis)

Attribute	Definition
Population	Adults living with HIV, smoking one or more tobacco cigarettes per day
Treatment condition	
Intervention	Offer of a preference-based smoking substitute menu (consisting of e-cigarettes, nicotine pouches and nicotine patches) free of charge for the participants.
Control	Standard smoking cessation practice in the SHCS
Endpoint	Self-reported abstinence from tobacco smoking in the past 7 days after 6 months
Summary measure	Odds ratio between groups, adjusted for key prognostic baseline covariates
Handling of intercurrent events	See table 2

Table 2: Definition and handling of intercurrent events for the primary estimand/analysis

Intercurrent event (type)	Strategy	Explanation
Death (truncating event)	While-alive strategy	Only outcomes before the occurrence of the death are considered.
Non-uptake of any smoking cessation product in the intervention group and uptake of an intervention smoking cessation product in the control group (treatment-modifying event)	Treatment policy strategy	The occurrence of these intercurrent event is part of the treatment condition. Participants stay in the intention-to-treat analysis set as randomized at baseline regardless of (non-)uptake thereafter.
Stop or switch of use of s smoking cessation product at any time during the intervention due to any reason (treatment-modifying event)	Treatment policy strategy	The occurrence of the intercurrent event is part of the treatment condition. Participants stay in the intention-to-treat analysis set, regardless of their adherence to the intervention.
Attrition from SHCS (truncating event)	Principal stratum strategy	Participants dropping out of the SHCS are excluded from the analysis set, assuming missing completely at random (no common cause between outcome and attrition), occurring at similar frequency across both groups and considered as such in the power calculation

Primary objective: The purpose of this trial is to test the effectiveness of offering a menu of different nicotine substitute products (e-cigarettes, nicotine pouches, nicotine patches in addition to usual care) for smokers in the SHCS in terms of self-reported tobacco cigarette abstinence at 6 months.

Secondary objectives:

- To assess the effect of offering the preference-based smoking cessation intervention on tobacco smoking cessation at 12 and 24 months.
- To assess the effect of offering the preference-based smoking cessation intervention on (i) reduction of smoked tobacco cigarettes per day; (ii) reduction of cardiovascular risk and (iii) occurrence of cardiovascular events or death.

Explanatory and implementation objectives:

- To investigate preferences and uptake of the different smoking cessation tools across various sociodemographic factors.
- To evaluate the safety and side-effects of nicotine pouches

3.2 Primary and secondary endpoints

The primary endpoint was chosen based on a smoking cessation core outcome set,[70,71] previous smoking cessation trials,[47] discussions with the Participant Advisory Board (see section 4.8), and the availability of routinely collected data in the SHCS. Secondary endpoints will generate evidence on long-term smoking cessation rates and broader health outcomes. Results from large scale randomized trials indicate that relative estimates from 7-day self-reported abstinence rates are similar to biochemically verified abstinence rates.[42,44,64] Moreover, the self-reported primary outcome will minimize missing and incomplete outcome data, because biochemical validation additionally requires urine or blood samples and a biobanking consent from participants.

Of note, by design (see section 3.3 about the TwiCs design), all trial endpoints (randomized comparisons) are routinely collected data, measured at cohort visits of the SHCS, regularly taking place approximately every 6 months. For the endpoint assessments at six months (180 days), a range of 120 to 270 days applies. For the endpoint assessments at twelve months, a range of 271 to 450 days after enrolment applies. If several measurements within the time window are available, the one closest to 6 months will be used. Additionally, we will collect information on the safety and usage of the interventional products via retrospective surveys only in the intervention group (see section 4.2.2).

Primary endpoint

- Tobacco smoking status (yes/no) measured as self-reported abstinence in the last 7 day at 6-month visit (window: 120-270 days)

Secondary endpoints

- Tobacco smoking status (yes/no) measured as self-reported abstinence in the last 7 day at 12-month visit (window: 271-450 days) and 24-month visit (window: 630-810 days).
- Mean change in the self-reported number of tobacco-based cigarettes smoked per day at 6,12, and 24 months.
- Self-reported use of any nicotine containing product other than tobacco cigarettes (yes/no). If yes, self-reported use of e-cigarettes (yes/no) or nicotine pouches (yes/no) or patches (yes/no) or other (yes/no) after 6 (window: 120-270 days), 12 (window: 271-450 days) and 24 (window: 630-810 days) months.
- Self-reported abstinence of any nicotine containing product (yes/no) after 12 (window: 271-450 days) and 24 (window: 630-810 days) months.
- Mean change in High Density Lipoprotein (HDL) and Low Density Lipoprotein (LDL) Cholesterol (mmol/l) at 6,12, and 24 months.
- Mean change in systolic and diastolic blood pressure (mmHg) at 6,12, and 24 months.
- Mean change in body weight (kg) from baseline to 6,12 and 24 months.
- Mean change in SCORE2[72]-risk prediction algorithm at 6, 12, and 24 months.
- Occurrence of cardiovascular events (myocardial infarction, coronary angioplasty/stenting, coronary artery by-pass grafting, carotid endarterectomy, stroke, deep vein thrombosis, pulmonary embolism, heart transplantation) and all-cause death at 6,12, and 24 months.

Safety endpoint

- Serious Adverse events (SAEs) are collected after 6, 12, and 24 months and are described in section 6.2.

3.3 Study design

We plan to conduct a multicenter, national, open-label, superiority, two-arm, randomized controlled trial using the TwiCs design approach, embedded in the SHCS. At least seven SHCS centers will participate (University Hospital Basel, University Hospital Bern, University Hospital Geneva, University Hospital Lausanne, Cantonal Hospital St. Gallen, University Hospital Zurich, and Cantonal Hospital Aarau). TwiCs is a relatively novel randomized trial design that was first described in 2010.[52] The overarching idea is to integrate a pragmatic randomized trial into an observational cohort study with randomization consent of participants to gain efficiency and mimic clinical practice. To do so, TwiCs uses a multistep consent procedure.

The consent procedure includes three levels. The first consent level is to collect longitudinal data as part of the SHCS ("**cohort consent**"). A signed cohort consent is a requirement to be eligible for the RETUNE trial. On a second level, cohort participants are asked for their consent to be randomized into future, not yet defined randomized comparisons ("**randomization consent**"). These first two consent steps are integral parts of the SHCS under the newly amended SHCS protocol (BASEC-Nr. 2023-02080, approved 05.07.2024) and currently the collection of the randomization consent is underway.

A signed randomization consent is also a requirement to be eligible for the RETUNE trial. If participants are randomized into the intervention group, they will be informed about being selected for the RETUNE trial and they can accept or decline the offered intervention ("**intervention consent**"). Participants randomized to the control group are not approached and will receive usual care according to the cohort procedure. If participants randomized into the intervention group accept the offered intervention (they can select one nicotine substitute product), they document their decision in written form ("intervention consent") and receive the products. The whole recruitment process including checking for eligibility will be integrated in the SHCS and all needed information is routinely collected data.

Figure 3: TwiCs design figure

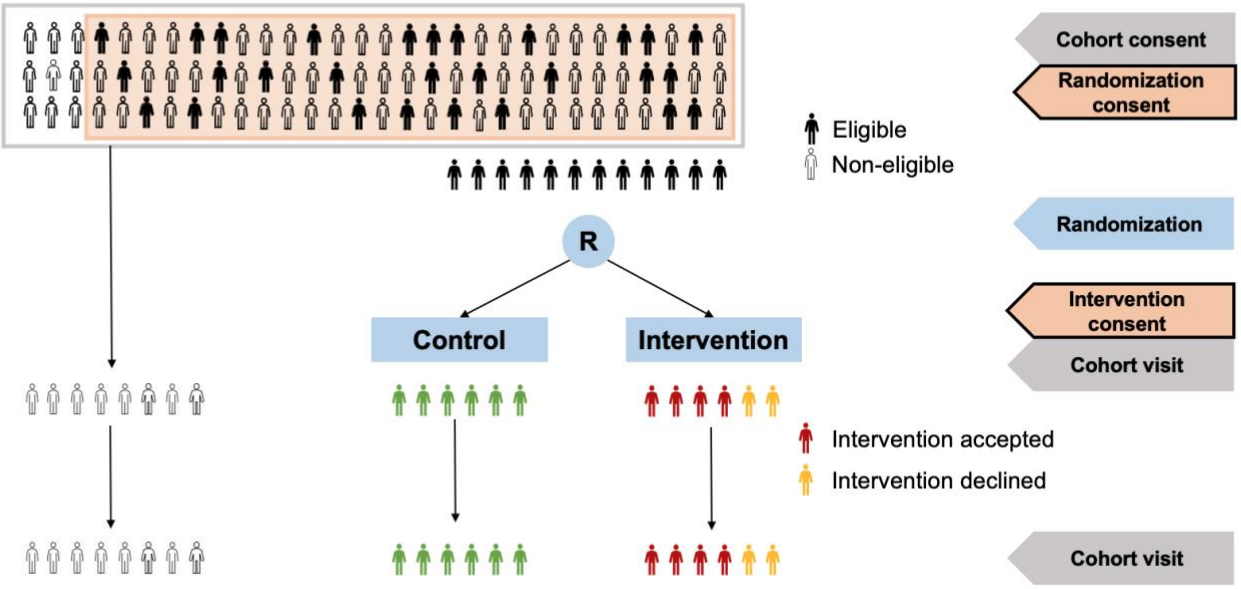

To be part of the SHCS, all participants need to sign the "cohort consent". Since the amendment of the SHCS protocol in August 2024 participants are also routinely asked for an additional "randomization consent". Smokers who signed the "randomization consent" and without exclusion criteria will become eligible for the RETUNE trial. They are randomized in a 1:1 ratio. Participants in the intervention group may accept or decline the offered intervention. If they

accept the intervention, they will sign an "intervention consent". The primary analysis will be conducted using an intention-to-treat analysis set. All trial endpoints are collected within the routine cohort visits.

3.3.1 Allocation concealment and blinding

The SHCS physicians who perform the randomization have no access to the random allocation sequence. The SHCS physicians who offer the intervention and collect the outcomes are aware of the group assignment. Participants in the control group are not aware of the trial (as per TwiCs design) Participants in the intervention are aware of the trial and their allocation to the intervention.

The primary endpoint will be self-reported and entered in the eCRF by the treating physician as part of routinely collected data during SHCS visits.

The data analyst who prepares the data and runs the analysis is aware of the group allocation.

3.3.2 Randomization

Cohort participants who have consented to be randomized to future interventions (see section 4.2.2) and meet the inclusion and exclusion criteria of the study will be assigned in a 1:1 ratio to the intervention and control groups. We will use a dynamic procedure using a stochastic treatment allocation algorithm based on the variance method to minimize imbalances simultaneously for region (French vs German speaking part of Switzerland), men having sex with men (yes/no), current drug user (yes/no), number of cigarettes smoked per day.[73] These variables – seen as the most important predictors of smoking cessation - were selected based on routine data availability and completeness, and expert knowledge.

The randomization will be conducted centralized via the SCHS data collection tool, by the SHCS data center located at the University Hospital Zurich.

4 STUDY POPULATION AND STUDY PROCEDURES

4.1 Inclusion and exclusion criteria, justification of study population

The RETUNE trial will include 972 PLWH who smoke tobacco cigarettes from the SHCS. Details on the sample size calculation are provided in section 5.1.

According to its pragmatic design, RETUNE will use broad and inclusive eligibility criteria, to ensure a high level of generalizability of the results for similar populations.

4.1.1 Inclusion criteria

- Signed informed consent for the data collection and participation in the SHCS (Cohort consent).
- Signed informed consent to be randomized to future interventions (Randomization consent).
- Age 18 years or older.
- Smoked one or more tobacco cigarettes per day (smoking status = yes) at the time of enrolment.

4.1.2 Exclusion criteria

- Currently using e-cigarettes or nicotine pouches or nicotine patches.
- Pregnant women.

4.1.3 Justification of the study population

As described in the background, PLWH have an increased risk for CVD and cancer, driven by life-style factors such as tobacco smoking. In Switzerland, PLWH are more likely to smoke tobacco cigarettes than people not living with HIV.

4.2 Recruitment, screening, and informed consent procedure

4.2.1 Screening and recruitment

RETUNE will use the SHCS as a platform to recruit participants according to the TwiCs design. All SHCS participants who have signed an informed consent to participate in future TwiCs (randomization consent, see section 4.2.2) will be screened for eligibility.

The eligibility criteria for the trial will automatically be checked using routinely asked questions within the SHCS visit procedure. During the consultation, the treating physicians (or a delegate) routinely ask SHCS participants about current smoking habits. If the tobacco smoking status is confirmed and no exclusion criteria are present, the participant will be eligible for the trial. On a separate page of the cohort data collection tool, the treating physician (or a delegate) is then prompted to press a button to randomize the participant into the intervention or control group. A randomization module is integrated in the SHCS data collection tool and will be set up by a data manager not involved in the trial. More details on the randomization process are available in section 3.3.2.

4.2.2 Informed consent

Informed consent is obtained in three different steps (cohort consent, randomization consent, intervention consent) according to the TwiCs design.

The "cohort consent" (to be part of the SHCS and its data collection) and "randomization consent" (to be randomized into future trials using the TwiCs design) are part of the SHCS protocol which was recently approved (BASEC-Nr. 2023-02080, approved 05.07.2024)

All eligible participants (tobacco smoker, cohort consent and randomization consent signed, and no exclusion criteria present) are randomized either to the control group or the intervention group. In the case of allocation to the control group, participants will receive standard smoking cessation counselling as part of routine care.

In the case of allocation to the intervention group, the treating physician (or a delegate) will offer the menu of the different nicotine substitute products to the participant. The participant will be encouraged to choose and try one of the products. If the participant is willing to accept any of the products, the physician (or a delegate) will briefly explain the usage, the planned duration of the intervention, and potential side-effects of the respective product and the participant will sign an additional informed consent form ("intervention consent"). The treating physician (or a delegate) will document the uptake of the respective interventional product. The "intervention consent" form will be signed and dated by the investigator or his designee at the same time as the participant signs. A copy of the signed informed consent will be given to the participant. The consent form will be retained as part of the trial records. Participants are informed that they can be contacted by the RETUNE trial team exclusively for the purpose of this trial. They are asked to provide their phone number and, if preferred, their email address. The contact details will only be used for the purpose of the trial (supply of interventional products, collection of adverse events of special interest, and retrospective reported use of interventional products) and deleted afterwards (see section 8.3 for details). Participants are told that they can withdraw from the intervention at any

time. However, they remain part of the SHCS and its data collection unless they also withdraw cohort participation.

4.3 Study intervention

The intervention consists of a menu of different nicotine substitute products from which trial participants can choose in the cohort consultation with the treating physician. Available are e-cigarettes, nicotine pouches, and nicotine patches, which are described in more detail below. All products are provided free of charge for 24 weeks. The participants will also receive an information brochure, how to use the product, which side effects should be reported, and how they receive supply.

4.3.1 Description of electronic nicotine delivery system (e-cigarettes)

This trial will use the Aspire© pod system OBY. The OBY e-cigarette will come in a user packet with a 500 mAh internal Li-Po battery, a fix integrated 1.2-ohm atomizer with 2ml capacity, a USB-C cable, and an instruction manual in French, German, Italian, English, Spanish and Dutch. The device is authorized for sale in Switzerland. The devices will be ordered via aspire-shop.de and fourtwenty.ch. Upon inclusion in the RETUNE trial, the devices will be given to the trial participants, and they will be informed about their use. Study nurses and treating physicians at the respective centers can provide additional support for the handling of the products.

We will use e-liquids from the company Gaiatrend© in France (alpha liquids). The liquids will be available in two different flavors (classic, menthol) and different nicotine concentrations (0 mg/ml, 3 mg/ml, 6 mg/ml, 12 mg/ml, 16 mg/ml). E-liquids are ordered via fourtwenty.ch and freevap.ch. The liquids were already tested in pre-clinical studies and used in a large-scale, multicenter, clinical trial in Switzerland and have proven to be safe.[64]

4.3.2 Description of nicotine pouches

We will use the Edelsnus© for this trial. We will have two different flavors with different nicotine content on offer (cold, 20 mg/g and onyx, 25 mg/g). The product is distributed by the Swiss company Edelsnus© and we will buy it via snushus.ch. Edelsnus are produced by the company MultiFill AG, which does not belong to any international tobacco company. The product is authorized for sale in Switzerland.

4.3.3 Description of nicotine patches

The nicotine patches (traditional nicotine replacement therapy) will be purchased from the company Nicotinell©. The nicotine patches are available in three different nicotine concentrations (21 mg, 14 mg, 7 mg). There are two different application schemes for heavy or moderate and mild smokers. The scheme for heavy smokers uses patches for a total of 12 weeks, reducing the dose of nicotine subsequently. The scheme for moderate and mild smokers lasts over a total of 9 weeks. These patches are classified as a drug, have been approved by Swissmedic (approval number 50582) and have been used in clinical practice for many years.

4.3.4 Comparator

This pragmatic trial will use the standard smoking cessation counselling in the SHCS as the comparator. To prevent contamination bias, the treating physicians will be trained not to offer the intervention menu (products) to the control group. At the smokers' request, further steps towards smoking cessation can be initiated, such as prescribing medication or referring them to special consultations. Of note, no interventional products from the trial will be dispensed to the control

group. Likewise, participants are not directed to use e-cigarettes or nicotine pouches on their own costs. Standardization of the control group with a rigid protocol is not intended. The different approaches of the individual centers and physicians will continue to be applied.

4.4 Study procedures

4.4.1 General Setting

RETUNE will be embedded in the SHCS and the regular cohort visits. The cohort visits take place approximately every 6 months and involve laboratory and clinical monitoring of the HIV infection, its medication as well as any comorbidities or concomitant diseases and their treatment. Further, preventive measures of common diseases in PLWH are also offered during the visits. For more detailed information regarding SHCS cohort visits and data collection, we refer to the latest version of the SHCS protocol (BASEC-Nr. 2023-02080, version 6.1).

4.4.2 Trial procedures and data collection

Cohort of the SHCS will take place approximately every 6 months. *Table 3* provides an overview of the trial procedure.

According to the TwiCs design, most relevant baseline data (inclusive demographic information) and trial endpoints are covered by the SHCS routinely collected data. Additional data collection exclusively for the RETUNE trial is restricted to the intervention group. *Table 4* provides an overview of the data collection.

Table 3: Trial procedures

**Procedure**	**Baseline visit**	**4-week Pilot study[1]** **+/- 6 weeks**	**8-week supply[2]**	**16-week supply[2]**	**Outcome visit month 6** **+/- 6 weeks**	**Outcome visit month 12** **+/- 6 weeks**	**Outcome visit month 24** **+/- 6 weeks**
Enrollment							
Eligibility screening	x						
Randomization	x						
Offering of intervention	x						
Informed consent ("intervention consent")	x						
Intervention							
Opportunity to switch products[3]		x	x	x			
Intervention	→	→	→	→	→		
Control	→	→	→	→	→		
Data collection							
Routine data collection	x				x	x	x
RETUNE-specific additional data collection in the intervention group		x			x		

[1] Only among the 30 participants included in the internal pilot study.

[2] Only among participants randomized to the intervention group who accepted an intervention product.

[3] These data are only collected in the intervention group

Table 4: Data collection

The routinely collected data used for RETUNE are marked in green. Data additionally collected for RETUNE are marked in orange.

Procedure	Baseline visit	4-week Pilot study[1]	Outcome visit month 6	Outcome visit month 12	Outcome visit month 24
Visit window		+/- 2 weeks	+/- 6 weeks	+/- 6 weeks	+/- 6 weeks
Smoking status (yes/no)	x		x	x	x
Number of cigarettes per day	x		x	x	x
Use of e-cigarettes (yes/no)	x		x	x	x
Use of other nicotine containing products (Nicotine patches, Chewing gums, Snus, Nicotine pouches, Pipe, Snuff, IQOS)	x		x	x	x
Cannabis use	x		x	x	x
Body weight	x		x	x	x
LDL-, HDL-, and total Cholesterol	x		x	x	x
Blood pressure	x		x	x	x
Cardiovascular event[2]			x	x	x
Safety assessment (SAE collection)[3]		x	x	x	x
Adverse event of special interest collection (see section 6.2.2)[4]		x	x		
Retrospective assessment of the product use (quantity of use)[4]			x		
Uptake and acceptability of the different components of the offered menu[4]	x	x			
Usage experience (quality of use)[4]		x	x		

[1] Only in the subgroup of participants included in the internal pilot study.

[2] Myocardial infarction, coronary angioplasty/stenting, coronary artery by-pass grafting, carotid endarterectomy, stroke, deep vein thrombosis, pulmonary embolism, heart transplantation

[3] A serious adverse event is any untoward medical occurrence that results in death or is life-threatening, requires in-patient hospitalization or prolongation of existing hospitalization, results in persistent or significant disability or incapacity, or causes a congenital anomaly or birth defect.

[4] These data are only collected in the intervention group

4.4.3 Storage conditions and supply of intervention products

The e-cigarettes (Aspire© pod system OBY) are purchased via aspire-shop.de and fourtwenty.ch. The e-liquids are purchased fourtwenty.ch and feevap.ch. The nicotine pouches are purchased via snushus.ch. By purchasing all products independently from the producers, we want to avoid conflicts of interest. The products are stored in the respective SHCS centers in a room with restricted access.

Trial participants will be instructed to store the products out of reach of children and pets. At the end of the study period, unused products may be retained by the trial participants.

In the case of a defective e-cigarette, a replacement product can be obtained from the SHCS center or from the Division of Clinical Epidemiology Basel.

A starter package of the chosen product (e-cigarettes, nicotine pouches or nicotine patches) will be given to trial participants during the inclusion visit. This should cover the required quantity for 8 weeks. After 6 weeks, the trial participants will receive a phone call or email (whatever they prefer). The participants are asked if they want to continue the product, switch to another product or stop their participation. If they do not actively decline, for example if we don't reach them in the time window, we will send a new supply for another 8 weeks. After 16 weeks, we will contact the participants again for the final delivery. They can decline the delivery, but this time they have not the possibility to switch the product again. Finally, the participants will receive the products for a total of 24 weeks free of charge. If there is a problem with a product between the fixed deliveries, the participants will receive a replacement product after contacting the trial team via phone or email or reach out to their treating physician.

To facilitate this supply chain, the participants will be asked to provide their phone number, e-mail address and postal address when entering the trial (part of the intervention consent). The information will be recorded in "RETUNE intervention group form" in REDCap®. The access to this information will be restricted to the RETUNE core team at the Division of Clinical Epidemiology Basel. All contact information will be deleted after completion of the trial. See section 8.3 for more detail.

4.5 Withdrawal and discontinuation

The trial intervention may be discontinued at any time. Participants who discontinue the trial intervention continue regular cohort visits within the SHCS.

4.6 Compliance with the intervention

Study personnel who recruit trial participants will be trained on the delivery of the intervention and of the potential benefits and harms of the products. The handling of e-cigarettes and nicotine pouches will be explained to the participants in a structured manner. They will also be made aware of possible side effects (see section 6.2.2.). To increase the compliance of the trial participants and to create an intervention that is as close to reality as possible, they can choose between the different nicotine replacement products. Furthermore, they can choose between different flavors of e-liquids and nicotine pouches.

The use of the administered products will be monitored retrospectively after the trial period using a survey.

Cross-over use of the interventional products in the control group will be monitored in the routine data collection of the SHCS.

4.6.1 Compensation for participants

No financial compensation or payments will be given to the participants.

4.6.2. Compensation for participating trial centers

The RETUNE trial will create additional work for the treating physicians and for the participating SHCS centers. This is compensated with a fixed fee of 105 CHF per included participant. In addition, this is intended to create an incentive for the trial centers to participate.

4.7 Participation in other clinical trials

Participants of the RETUNE trial are allowed to participate in other observational and interventional studies in the SHCS if there is no interference with the intervention or outcomes investigated in RETUNE. The scientific board of the SHCS and the RETUNE study team will ensure this.

4.8 Patient and public involvement and the participant advisory board

Based on the guiding principle of patient and public involvement (PPI)[74], this trial was planned together with patient representatives from the SHCS, collaborating with them in every phase of our trial. As a first step, we have conducted informal interviews with three patient representatives (2 men, 1 woman) from the German and French speaking regions of Switzerland and we have discussed and incorporated their initial thoughts and comments on the study design, the intervention, the study procedures, and the study endpoints. We have drafted a PPI agreement (based on the Swiss Clinical Trial Organization template[75]) to outline responsibilities, expectations, timelines as well as remuneration details to formalize the PPI and establish a formal Participant Advisory Board for this project. To provide more details, the agreement is available as a supplement to this protocol. The PPI will be reported using the *GRIPP2* reporting guidelines in the final manuscript.[76]

5 STATISTICS AND METHODOLOGY

5.1 Sample size calculation

We assume a smoking cessation rate of 8.5% in the control arm (based on SHCS data), a 20% cessation rate in the intervention arm (based on external evidence of similar trials[41-43]), and an attrition rate of 3% (based on SHCS data).[13]

Taking into account a low uptake of only 50% in the intervention and thus dilution of the intervention effect, we aim at enrolling a total 972 participants (486 in each arm) to achieve 80% power for a two-sided alpha level at 5%. We will update our sample size during recruitment depending on observed uptake rates, monitored at pre-defined recruitment timepoints.

The full R code for the sample size calculation is available on GitHub (https://github.com/alainamstutz/TwiCs_samplesize/blob/main/TwiCs_samplesize.md)

Due to the integration of the trial in the SHCS, eligibility of cohort participants can be pre-assessed. *Table 7* shows the current situation regarding smoking behavior in the SHCS. The SHCS includes 9470 PLWH under active follow-up, of which 4873 are under treatment in one of the RETUNE trial centers (Data from December 05, 2022). At the last follow-up visit, 1576/4873

(32.3%) PLWH reported smoking tobacco cigarettes. The median number of tobacco cigarettes smoked per day was 12 (IQR = 7 -20).

Table 5: Eligible participants for RETUNE

Center	Smoking	Non-smoking
Zurich	541	1171
Basel	205	394
Bern	232	496
Geneva	115	319
Lausanne	272	588
St. Gallen	211	329
Total	1576	3297

5.2 Adaptive readjustment

The uptake of the offered intervention plays a key role in the sample size calculation of trials using the TwiCs design. We start with a conservative scenario of 50% uptake. As per guidance from the literature and conducted by other TwiCs, we will re-evaluate this critical assumption at pre-specified timepoints (after 100, 200, and 300 randomized participants).[55,77] If the uptake is higher than expected, we will reduce the target total sample size (see *figure 4* below for the correlation of uptake and the needed sample size). Since we will not analyze outcomes at the sample size re-evaluation time points, these are not formal interim analyses with alpha spending and no correction for multiplicity is needed. If uptake falls well below 45%, we will include more sites or decide to discontinue based on available budget and operational resources.

Figure 4: Influence of effect size on target sample size

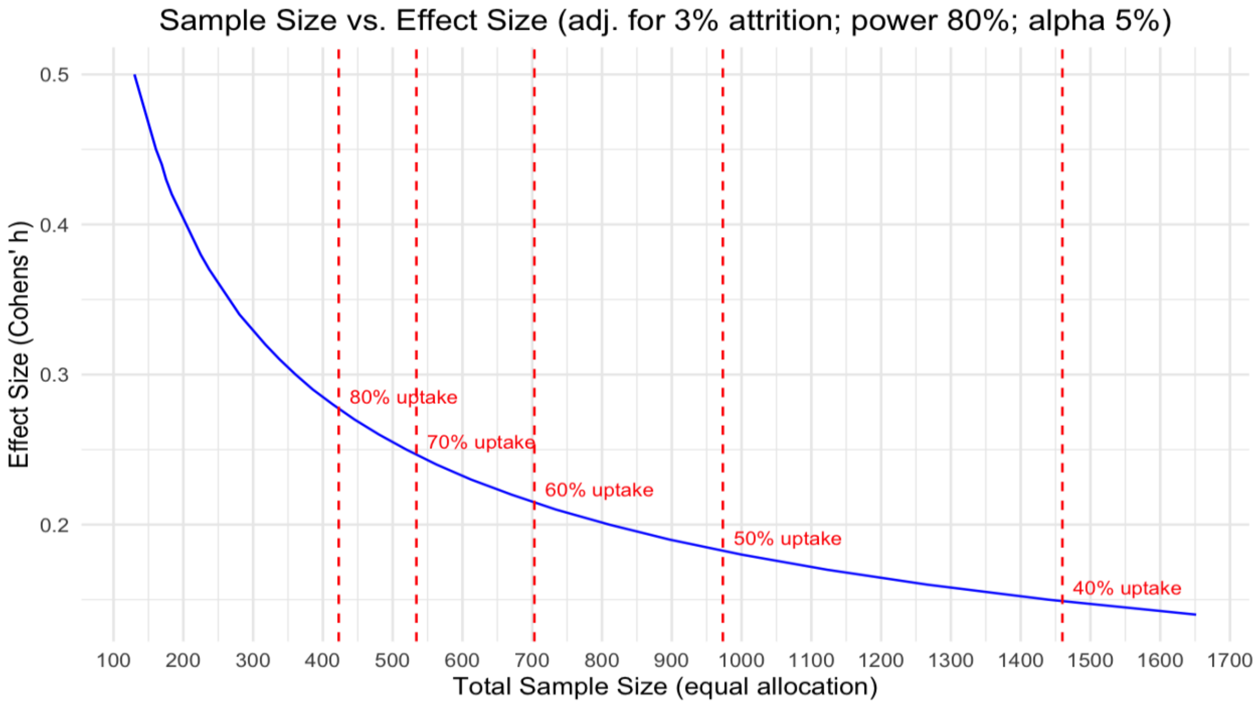

The effect size is influenced by the uptake of the offered intervention. If the uptake is lower, then the effect size becomes exponentially smaller, influencing the target sample size. For our calculation we used a two-

sided alpha of 5%, a power of 80%, and an attrition rate of 3%. A Cohen's effect size of 0.18 corresponds to a cessation rate of 8.5% in the control group and a diluted intervention effect of 14.25% (instead of 20%) in the intervention group, accounting for an uptake of 50% in the intervention group.

5.3 Internal pilot study

Pilot studies are a subset of feasibility studies in which the feasibility of an RCT, or a sub-aspect of an RCT, is tested on a usually smaller scale, i.e. with fewer participants.[78] The goal of a pilot study is to evaluate important logistical aspects of a future trial and optimally integrate them into the trial design. This helps to avoid errors in trial conduct and makes pilot studies a valuable contribution to optimizing the internal validity of high-quality clinical trials.[79] Two types of pilot studies can be distinguished, external pilot studies and internal pilot studies. In an external pilot study, the participants are excluded from the final outcome data set. Consequently, participants who would be eligible for the trial are no longer available. In an internal pilot study, participants and their outcome data can be transferred from the "pilot phase" to the main trial with its final analysis.[80]

The internal pilot study will include the enrolment of at least 30 people randomized to the intervention group which are accepting the offered intervention. The internal pilot study will recruit participants at the center in Basel and in at least one other center.

The pilot study will investigate logistical challenges and generate knowledge about the popularity of the different components (nicotine patches, e-cigarettes, nicotine pouches) of the preference-based smoking cessation menu. Based on this, we can tailor our supply. After 30 days, the pilot participants of the intervention group will be interviewed by telephone about their use behavior. We will offer two to a maximum of three different flavors and nicotine concentrations of e-cigarettes and nicotine pouches, which will be further tailored according to their popularity after the pilot study phase. Details regarding the criteria studied during the pilot phase are listed in *Table 3*. If we observe major concerns regarding the feasibility of the trial during the internal pilot study, we will reconsider the continuation of the trial.

Table 6: Aims of the internal pilot study

Aim	Measurement	Explanation
To assess the safety of the interventional products	After 30 days, the participants in the intervention group will be asked if they experienced any safety issues (Adverse events of special interest, see also section 6.2.2) with the products.	The tested intervention products are of low risk for the participants, since they are all available on the free market. We will test this assumption and modify the offered menu if needed.
To assess the uptake, acceptability/preference of the different components of the intervention package.	Uptake of the different components of the offered menu measured.	Based on this information, we will adapt the supply of products. For example, if e-cigarettes should be more popular than nicotine pouches, we will order more e-cigarettes for the full trial.

To assess the preferences regarding the different products and flavors and the experience of the participants regarding the handling of products.	After 30 days, the participants in the intervention group will be asked regarding their usage experiences of the different products and their preferences for flavors of nicotine pouches and e-cigarettes.	We are interested in the experiences of the participants with the respective products. Moreover, nicotine pouches and e-cigarettes are available in different flavors. The collected knowledge will help us to further optimize the offered products and better instruct the participants reading the usage.

5.4 Description of statistical methods

The analysis of the trial will be conducted at the Division of Clinical Epidemiology at the University Hospital Basel. Before the recruitment will be completed, a statistical analysis plan will be written, detailing the steps of data processing and statistical analyses.

All analyses will be performed using Stata[81] or R[82].

5.4.1 Dataset to be analyzed

The primary analysis set will be an intention-to-treat analysis set. It will include all randomized participants as they were randomized (see also definition of primary estimand in section 3.1). Participants randomized to intervention are analyzed as such, i.e., regardless of product uptake. Participants randomized to the control group are analyzed as such, i.e., regardless of any intervention product use outside of the study (cross-over).

5.4.2 Primary analysis

The primary outcome will be assessed in the intention-to-treat set as defined in section 5.4.1., using a logistic regression model adjusted for the variables used in the minimization (region [French vs. German speaking part of Switzerland], men having sex with men [yes/no], current drug user [yes/no], number of cigarettes per day) and will be reported as an adjusted odds ratio with 95% Wald confidence interval.[83-85] Superiority of the intervention vs control will be assessed according to the two-tailed p-value of the intervention regression coefficient and an alpha level set to 0.05. Additionally, absolute risk difference and proportions of smoking cessation under intervention and control will be estimated using marginal standardization, with 95% percentile interval calculated from 1,000 bootstrap samples. Hypothesis testing will only be conducted for primary.

5.4.3 Secondary analyses

Table 7 summarizes the statistical analysis of the secondary endpoints. For the secondary outcomes we will use the same model as for the primary outcome but use linear regression in case of continuous outcomes and add baseline adjustment of the outcome where appropriate. The secondary outcomes will be reported with 95% confidence intervals to support descriptive interpretation and are of exploratory nature. Further details are provided in the statistical analysis plan.

Table 7: Statistical analysis of secondary endpoints

Tobacco smoking status (yes/no) measured as self-reported abstinence in the last 7 days at 12-month visit (window: 300-420 days) and 24-month visit (window: 650-800 days).	Logistic regression model adjusted for the variables used in the minimization (region [French vs. German speaking part of Switzerland], men having sex with men [yes/no], current drug user [yes/no], number of cigarettes per day) and will be reported as an adjusted odds ratio with 95% confidence interval.
Mean change in the self-reported number of tobacco-based cigarettes smoked per day at 6,12, and 24 months.	Linear regression model adjusted for the variables used in the minimization (region [French vs. German speaking part of Switzerland], men having sex with men [yes/no], current drug user [yes/no], number of cigarettes per day) and will be reported as an adjusted odds ratio with 95% confidence interval.
Self-reported use of any nicotine containing product other than tobacco cigarettes (yes/no). If yes, self-reported use of e-cigarettes (yes/no) or nicotine pouches (yes/no) or patches (yes/no) or other (yes/no) after 6 (window: 120-270 days), 12 (window: 271-450 days) and 24 (window: 630-810 days) months.	Logistic regression model adjusted for the variables used in the minimization (region [French vs. German speaking part of Switzerland], men having sex with men [yes/no], current drug user [yes/no], number of cigarettes per day) and will be reported as an adjusted odds ratio with 95% confidence interval.
Self-reported abstinence of any nicotine containing product (yes/no) after 12 (window: 271-450 days) and 24 (window: 630-810 days) months.	Logistic regression model adjusted for the variables used in the minimization (region [French vs. German speaking part of Switzerland], men having sex with men [yes/no], current drug user [yes/no], number of cigarettes per day) and will be reported as an adjusted odds ratio with 95% confidence interval.
Mean change in HDL-, LDL- and total Cholesterol (mmol/l) at 6,12, and 24 months.	Linear regression model adjusted for the variables used in the minimization (region [French vs. German speaking part of Switzerland], men having sex with men [yes/no], current drug user [yes/no], number of cigarettes per day) and will be reported as an

	adjusted odds ratio with 95% confidence interval.
Mean change in systolic and diastolic blood pressure (mmHg) at 6,12, and 24 months.	Linear regression model adjusted for the variables used in the minimization (region [French vs. German speaking part of Switzerland], men having sex with men [yes/no], current drug user [yes/no], number of cigarettes per day) and will be reported as an adjusted odds ratio with 95% confidence interval.
Mean change in body weight (kg) from baseline to 6,12 and 24 months.	Linear regression model adjusted for the variables used in the minimization (region [French vs. German speaking part of Switzerland], men having sex with men [yes/no], current drug user [yes/no], number of cigarettes per day) and will be reported as an adjusted odds ratio with 95% confidence interval.
Mean change in SCORE2 prediction algorithm at 6,12, and 24 months.	Linear regression model adjusted for the variables used in the minimization (region [French vs. German speaking part of Switzerland], men having sex with men [yes/no], current drug user [yes/no], number of cigarettes per day) and will be reported as an adjusted odds ratio with 95% confidence interval.
Occurrence of cardiovascular events (myocardial infarction, coronary angioplasty/stenting, coronary artery by-pass grafting, carotid endarterectomy, stroke, deep vein thrombosis, pulmonary embolism, heart transplantation) at 6,12, and 24 months.	This endpoint will be presented descriptively.
Serious Adverse events (SAEs) are collected after 6, 12, and 24 months.	This endpoint will be presented descriptively.

Since we expect low uptake of the intervention products at baseline (currently estimated at 50%), the intention-to-treat estimand will give us an unbiased estimate for offering the intervention to all. While this estimate is of interest to policy makers, it might be less so for patients and treating physicians.[86]

We will apply two observational causal inference methods to inform possible per protocol effects of interest. As any statistical analysis, they rely on specific assumptions that need to be justified:

i) Estimation of the hypothetical per protocol “Effect of everyone had taken up a product at baseline vs no-one had taken up a product at baseline” (Average Treatment Effect)

a. This per protocol effect will be estimated using data generated by the trial. We will compare outcomes of trial participants that received the intervention with those from participants that did not receive intervention (i.e., participants in the control group and non-uptakers in the intervention group). We will generate these pseudo-populations using inverse probability of treatment weights to correct for potential imbalance of main confounders. Main confounders are the factors used for the minimization.

b. The main assumption for this analysis (which does not rely on randomization anymore) is the usual core assumption of any standard observational analysis, i.e., no unmeasured confounding, that means that all confounders are measured and are controlled for. See Directed Acyclic Graph (DAG) in *Figure 5*.

Figure 5: DAG – Confounding in observational analysis

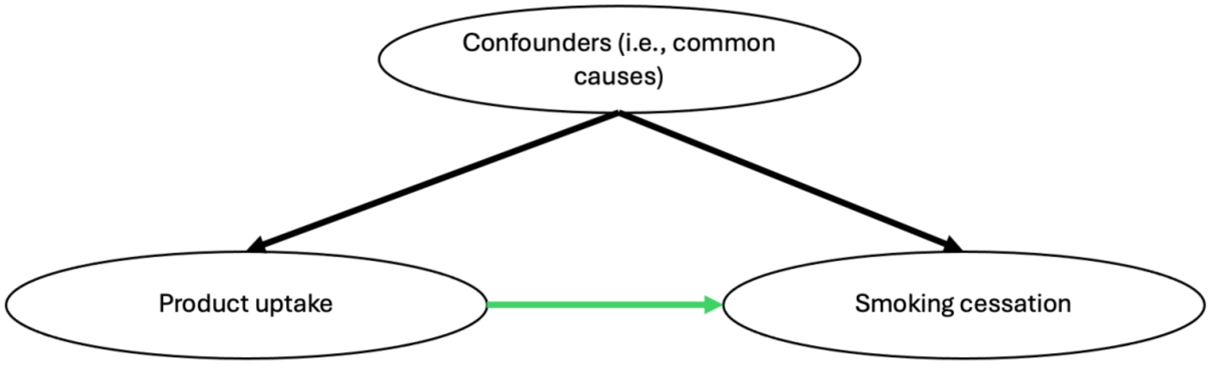

Notes: Confounders were defined based on expert knowledge: region (French vs German speaking part of Switzerland), men having sex with men (yes/no), current drug user (yes/no), Number of cigarettes smoked per day.

Green arrow: Average causal effect to be estimated.

Further details will be outlined in the statistical analysis plan.

ii) Estimation of the hypothetical per protocol “Effect of the intervention for participants who complied with the treatment assigned to”” (Complier Average Causal Effect).

a. This per protocol effect will be estimated using an instrumental variable approach.

b. While instrumental variable methods do not rely on the core assumption of no unmeasured confounding, they however rely on a different set of assumptions. First, the three core assumptions of the instrumental variable need to hold:

   i. “Randomization is associated with product uptake” (*relevance assumption*). This can be proven from the data and will undoubtedly be true in RETUNE since it is an RCT and randomization the instrument.

   ii. “The randomization and smoking cessation do not share common causes” (*independence assumption*). This can be proven from the data and will indubitably be true in RETUNE since it is an RCT and randomization the instrument.

iii. "Randomization does not affect smoking cessation except through the potential effect of the intervention" (*exclusion restriction assumption*). This cannot be shown empirically but is reasonable to hold since both groups receive standard counselling and suggested in other similar RCT settings.[87,88]

c. Second, to estimate the complier effect including a point estimate, a fourth assumption is needed. While the homogeneity assumption (the effect of the intervention on the outcome is exactly the same for all participants) is a rather strong assumption, we will choose instead the *monotonicity assumption*. The TwiCs design is a perfectly suited RCT design for this assumption to hold, because the control group is not aware of the intervention group and vice versa. In this sense, there is no possibility of defiers being present in the study population, meaning people who accept the intervention if not offered but deny the intervention if offered. This - plus the fact that TwiCs often have low uptake of their interventions - are the reasons why there is a prolific literature of using such methods as per protocol analyses in TwiCs.[89-92]

Figure 6: DAG– Randomization as instrumental variable

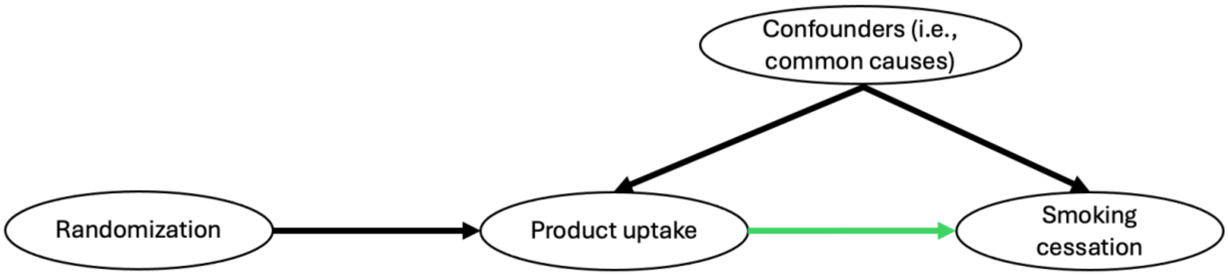

Notes: Since the effect of product uptake on smoking cessation is measured using randomization as the instrument, confounders are irrelevant.

Green arrow: Complier Average Causal Effect to be estimated.

Further details will be outlined in the statistical analysis plan.

5.4.4 Interim analysis

No interim analysis will be conducted.

5.4.5 Subgroup analysis

We plan exploratory subgroup analysis (no pre-specified hypothesis) for the baseline number of cigarettes smoked per day (continuous), age (continuous), and sex (binary). We will include corresponding interaction terms – one at a time - in the adjusted logistic regression model. If a p-value of an interaction term turns out to be smaller than 0.1, we will assess the credibility of the effect using the ICEMAN tool.[93] Further details will be outlined in the statistical analysis plan.

5.5 Handling of missing data and drop-outs

No missing covariates are expected since all adjusting factors are used for minimization and

therefore mandatory for randomization. Missing outcome data will be imputed using multiple imputation by chained equation techniques. Additionally, we will perform a sensitivity analysis where participants with missing outcomes will be excluded from the analysis (complete case analysis). Further details will be outlined in the statistical analysis plan.

6 REGULATORY ASPECTS AND SAFETY

6.1 Local regulations / Declaration of Helsinki

This study is conducted in compliance with the protocol, the current version of the Declaration of Helsinki[94], the ICH-GCP[95], the HRA[96] as well as other locally relevant legal and regulatory requirements.

6.2 (Serious) Adverse Events: Definitions, collection, and assessment

6.2.1 Adverse Events (AE)

An adverse event (AE) is any untoward medical occurrence in a patient which does not necessarily have a causal relationship with the trial procedure. An AE can therefore be any unfavorable or unintended finding, symptom, or disease temporally associated with a trial procedure, whether or not related to it.

6.2.2 Adverse Events of Special Interest (AESI)

Adverse events of special interest (AESI) are AEs probably related to the tested nicotine substitute products. We anticipate the following AESI, and RETUNE study team will ask them actively in a retrospective survey amongst participants in the intervention group (online or telephone based):

- New or increased nausea after use of the investigational product
- New or increased emesis after use of the investigational product
- New or increased headache after the use of the investigational product
- New or increased dizziness after the use of the investigational product
- New or increased respiratory symptoms: cough, phlegm, wheezing, sore throat after the use of the investigational product
- New or increased gingival pain after use of the investigational product
- New or increased gingival bleeding after use of the investigational product
- New or increased mouth ulcers after use of the investigational product
- New or increased eczema / allergic skin reactions after use of the investigational product
- New or increased mouth and tongue irritation / change of the oral mucosa after use of the investigational product

6.2.3 Serious Adverse Event (SAE)

A serious adverse event (SAE, ClinO, Art. 63)[97] is any untoward medical occurrence that

- Results in death or is life-threatening,
- Requires in-patient hospitalization or prolongation of existing hospitalization,
- Results in persistent or significant disability or incapacity, or
- Causes a congenital anomaly or birth defect

6.2.4 Collection of AE, SAE, AESI

As this is a pragmatic trial with a low-risk intervention, we propose a risk-based safety monitoring. SAEs will be collected in the routine data. AESI will be collected using a retrospective survey among the intervention group only. As such this will be a single-arm assessment without a randomized comparator and interpreted as such. We will not collect any other AEs.

6.2.5 Assessment of causality and severity

The causality assessment of all SAE will be performed by a trial physician using information from the retrospective survey in the intervention group and routinely collected data within the SHCS. An independent physician will provide a statement whether the SAE is definitely related, probably related, possibly related, unlikely related, or unrelated to the offered intervention.

Table 8: Causality assessment

Relationship	Description
Definitely	Temporal relationship<br>Improvement after dechallenge*<br>Recurrence after rechallenge<br>(or other proof of drug cause)
Probably	Temporal relationship<br>Improvement after dechallenge<br>No other cause evident
Possibly	Temporal relationship<br>Other cause possible
Unlikely	Any assessable reaction that does not fulfill the above conditions
Not related	Causal relationship can be ruled out
*Improvement after dechallenge only taken into consideration, if applicable to reaction	

An independent trial physician makes a severity assessment of the event as mild, moderate, or severe. Mild means the complication is tolerable, moderate means it interferes with daily activities and severe means it renders daily activities impossible.

6.2.6 Reporting of SAEs (see ClinO, Art. 63)[97]

All SAEs are documented and reported within a maximum of 24 hours after being aware of it to the Sponsor-Investigator.

If it cannot be excluded that the SAE is attributable to the intervention under investigation, the Sponsor-Investigator reports it to the Ethics Committee via BASEC within 15 days. A corresponding template from Swiss Ethics will be used.

6.3 (Periodic) safety reporting and reporting of general progress

All deaths and all other SAEs that are possibly or definitely related to the study intervention will be reported to the Ethics Committee of Northern and Central Switzerland (EKNZ) immediately (see section 6.2.6) and summarized on a yearly basis. The investigator also informs the Ethics Committee about the general progress of the clinical trial (ClinO, Art. 43).

6.4 Pregnancy

Pregnant women will be excluded from the trial. Pregnancy is usually collected as part of SHCS data collection. We will exclude pregnant women not because the intended intervention is particularly harmful in this population, especially compared to the known harms from tobacco smoking, but the ethics committee had concerns with nicotine replacement for pregnant women, and we followed the recommendation of the ethics committee.

6.5 Amendments

Substantial changes to the trial setup and organization, the protocol and relevant trial documents will be submitted to the Ethics Committee for approval before implementation. Under emergency circumstances, deviations from the protocol to protect the rights, safety and well-being of human subjects may proceed without prior approval of the Ethics Committee. Such deviations will be documented and reported to the Ethics Committee as soon as possible.

Substantial amendments are changes that affect the safety, health, rights and obligations of participants, changes in the protocol that affect study objective(s) or central research topic, changes of trial site(s) or of trial leader and sponsor (ClinO, Art. 29).

A list of substantial changes is also available on www.swissethics.ch.

A list of all non-substantial amendments will be submitted once a year to the competent EC together with the yearly safety report.

6.6 Notification and reporting upon completion, discontinuation or interruption of the study

After enrolment of the first participant and upon regular trial completion, the Ethics Committee is notified via the BASEC within 30 days (ClinO, Art. 38).[97] The sponsor-investigator may terminate the trial prematurely in case of:

- Ethical concerns
- Insufficient participant recruitment
- When the safety of the participants is doubtful

In case of premature trial termination or trial interruption, the Ethics Committee will be notified via BASEC within 15 days (ClinO, Art. 38).[97]

A final report is submitted to the Ethics Committee via BASEC within a year after completion or discontinuation of the trial, unless a longer period is specified in the protocol (ClinO, Art. 38).[97]

6.7 Insurance

In the event of study-related damage or injuries, the liability of the University Hospital Basel provides compensation, except for claims that arise from misconduct or gross negligence.

7 FURTHER ASPECTS

7.1 Overall ethical considerations

The purpose of this trial is to address the need to reduce tobacco smoking among SHCS participants. RETUNE follows the concept of harm reduction by substituting smoking tobacco cigarettes by less harmful alternatives.

We propose to test a pragmatic intervention using an innovative trial design to minimize the hurdle for the participants and the treating physician to participate in the trial. We will generate real-life evidence about making new nicotine substitute products available on a low threshold to all smokers, regardless of their willingness to quit smoking. This intends to change the way of smoking cessation in clinical trials as well as in clinical routine. In case the intervention proves to be effective, it can be directly transferred into clinical practice.

7.2 Risk-benefit assessment

The investigational intervention is considered low risk. The intervention is designed to help trial participants quit smoking tobacco with the overarching idea of harm reduction by substitution. In case the intervention is effective, trial participants will benefit directly by reducing their risk of tobacco-related cardiovascular disease and cancer. Similarly, the society and smokers in other settings will profit from improved evidence about smoking cessation. Finally, the evidence generated in this trial may inform national and international clinical guidelines as well as policymakers to improve smoking cessation strategies.

All interventions used in RETUNE (e-cigarettes, nicotine pouches and nicotine patches) are approved by the Swiss authorities and freely available. The nicotine patches, approved by the Swiss Agency of Medical Products (Swissmedic). All products may cause local reactions and irritation of the skin or mucous membrane and systemic reactions to the nicotine intake such as nausea and headache. We will try to minimize the risks associated with the incorrect application of the products by instructing the participants accordingly. The study participants will be informed to keep the products out of reach of children and pets.

Even though e-cigarettes and nicotine pouches are not under the jurisdiction of Swissmedic, we will determine adverse events of special interest, serious adverse events, and risks with the same accuracy as if they were therapeutic products.

7.3 Time plan and trial duration

Table 9: Overview trial duration

Year	2025				2026				2027			
**Quarter**	**1**	**2**	**3**	**4**	**1**	**2**	**3**	**4**	**1**	**2**	**3**	**4**
Recruitment of Participants for RETUNE												
Internal pilot trial												
Primary outcome assessment												
Secondary outcome assessment												
Analyzing data & manuscript writing and submission												
Long term follow-up with subsequent analysis												

We aim to start the recruitment in February 2025. SHCS follow-up visits take place every 6 months. We anticipate completing the recruitment by the end of August 2026. The recruitment will start at the SHCS center Basel. Subsequently, the other involved centers will open to reach the full recruitment capacity.

The primary outcome data will be collected at 6 months after inclusion during the following cohort visit. Considering the time of inclusion in the trial and a margin of 2 months, we expect to complete the collection of the primary endpoint by the end of February 2027.

The secondary outcomes data will be collected 12 months after inclusion in the trial. Considering the same margin of 2 months, we expect to complete the data collection at the end of August 2027. Given the outcome collection within the routine SHCS data collection, long term follow-up data (24 months after randomization) will be collected automatically without trial specific follow-up.

7.4 Process evaluation

RETUNE tests a complex intervention that involves offering a menu of different nicotine substitute products to participants in the SHCS who smoke tobacco cigarettes. To better understand how this intervention works, we are planning a process evaluation study. This study will use a mixed-methods design (combining qualitative and quantitative measures) to investigate the delivery (offer), uptake, adoption, and implementation of the intervention, as well as the contextual factors influencing these processes. The process evaluation study will be described in detail elsewhere.

8 QUALITY CONTROL AND DATA PROTECTION

8.1 Quality measures

The treating physicians of the participating SHCS centers will be trained by the RETUNE team regarding screening, recruitment, randomization procedure, consent procedures, and how to explain and deliver the intervention. We will emphasize the concept and importance of harm reduction in smoking cessation. This training will be conducted in person for each center

individually to take local differences into account. Moreover, the study team will provide training material and leaflets for the treating physicians and study nurses.

RETUNE will use the routinely collected data from the SHCS for most endpoints. Quality measures for the SHCS data collection are described in the SHCS protocol (BASEC-Nr. 2023-02080). Additional data on (S)AE and use of the chosen products will be collected retrospectively via surveys sent out by the RETUNE team. This information will be collected via REDCap and stored on a server at the University Hospital Basel.

The data records will be kept under protection to ensure the confidentiality of the trial participants. The principal investigators and their delegates will have access to the records. The principal investigators will permit access to the data records to regulatory agencies in case of audits.

No independent Data- or Safety Monitoring Committee is planned due to the low risk of the tested intervention. Safety monitoring will be conducted as outlined above in section 6.2.

8.2 Data recording and source data

RETUNE will use routinely collected data from the SHCS. The SHCS eCRF is a Django-based web-application hosted at the University of Zürich (https://www.djangoproject.com). The application is based on PostgreSQL (https://www.postgresql.org). The application was developed by the SHCS and includes an audit trail, which means that it is traced who changed which data and when. A daily backup is done.

Additional data like information on (S)AE, use of the individual products, and contact information of the participants to provide supply of the interventional products over the course of the trial will be recorded using REDCap (https://www.project-redcap.org/) surveys.

The signed intervention consent forms represent the only source data in the RETUNE trial. They will be stored at the respective centers. For data recording and export we will work closely together with the SHCS data center at the University of Zurich. Details regarding the eCRF, the record of data and the data management are described in the SHCS protocol (BASEC-Nr. 2023-02080).

8.3 Confidentiality and coding

At the inclusion cohort visit, the participants who accept the offered intervention are asked to provide their postal address and preferred communication channel (telephone number or email address). This information will be used to contact the participants for supply of the interventional products (see section 4.4.3) and to conduct retrospective surveys in the intervention group to collect intervention on potential SAEs and usage of the interventional products (see table 4 section 4.4.2). This information will be stored in a REDCap based case report form. The REDCap is hosted by the Department of Clinical Research at the University Hospital Basel. Only the principal investigators and their delegates will have access to this data.

When the participants sign the intervention consent, the treating physician writes by hand the REDCap identifier (unique for each participant) on the intervention consent sheet. The intervention consent sheets are stored at each cohort center. Using the signed consent sheets with the REDCap identifier, each center creates a decoding key (excel file) with the SHCS number and the REDCap identifier. This procedure ensures that identifying patient data (e.g. name, postal address) in combination with the SHCS identifier cannot be used outside of the respective SHCS center.

The information recorded in REDCap (stored at the University Hospital Basel) will be kept separate from the SHCS data collection (stored at the University of Zurich). After completion of the RETUNE trial, an anonymized data set with the results from the surveys, on SAE and usage of the products, will be exported from REDCap. No identifying patient data will be exported. This data set will then be merged with the routinely collected data from the SHCS using the de-coding key at each SHCS center.
This procedure ensures that identifying participant data leaves the SHCS center only for the purpose of the RETUNE trial and without the corresponding SHCS identifier.

On the SHCS eCRFs to collect the routine cohort data, participants are only identified by a unique participant number. The eCRFs are stored centrally on a server of the University Hospital Zurich. The SHCS eCRFs are password protected and only members of the SHCS have access. For detailed information we refer to the protocol of the SHCS. A list of participants identification will be stored in the investigator site file at each study site.

No biological material is collected as part of this trial. For more detailed information about protection, storage, shipping (including transport to other centers or other countries) and processing of routine biological material in the SHCS we refer to the protocol of the SHCS (regarding the biobanking). For shipping of biological material outside of the SHCS centers (if needed), separate transfer agreements will be signed.

Figure 7: Data collection, processing, export and decoding

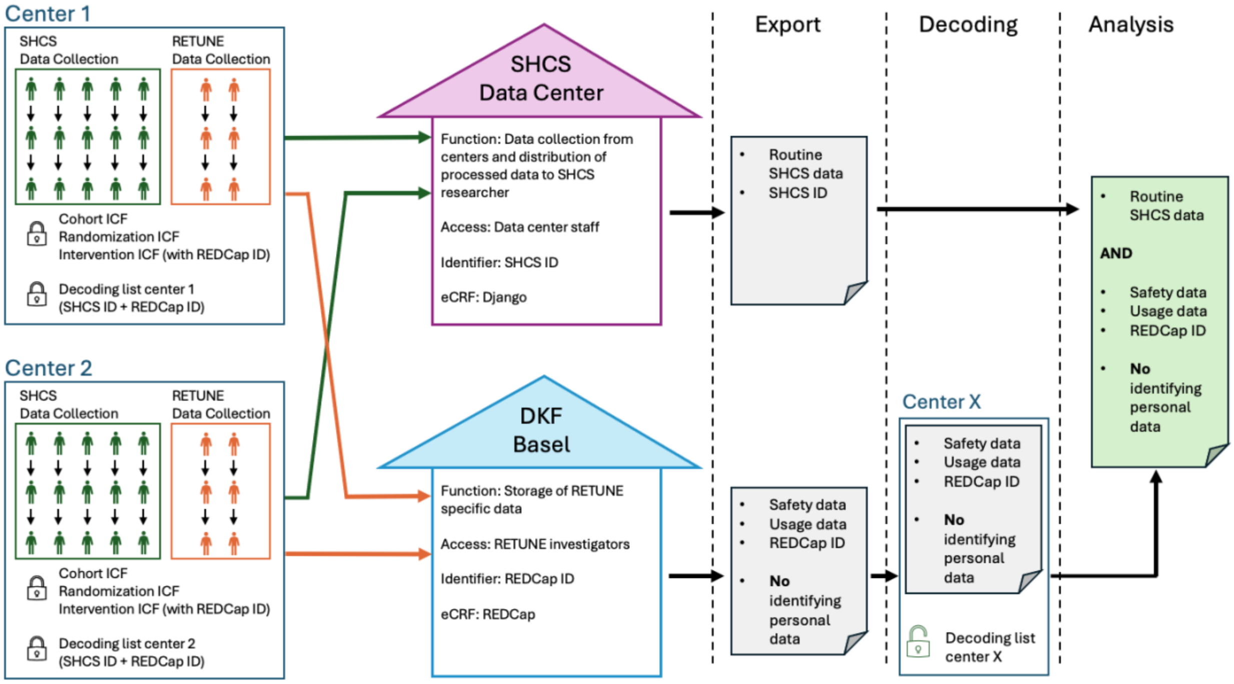

Routine SHCs data is collected at the different SHCS centers via Django and stored and processed centrally at the SHCS data center at the University of Zurich. Data additionally collected for RETUNE (including identifying personal data like phone number, Email address or postal address) is collected via REDCap and stored at the DKF at the University Hospital Basel. The decoding list which allows combining RETUNE data with SHCS data remains at the respective SHCS center. After completion of the trial, the RETUNE specific data is exported without identifying personal data. The decoding lists are used to combine the RETUNE data with the SHCS data at the respective centers.

DKF=Department Clinical Research, ICF=Informed Consent Form, ID=Identifier, RETUNE=Reduce Tobacco Use In People Living With HIV, SHCS=Swiss HIV Cohort Study

8.4 Retention and destruction of study data and biological material

At the end of the trial or in case of premature termination, all trial data will be locked and archived. Since most of the trial data are routinely collected within the SHCS, the data will be stored as described in the SHCS protocol on a server of the University of Zurich. Additional data not collected within the SHCS (retrospective surveys conducted via REDCap) are stored by the Department of Clinical Research at the University Hospital Basel on a secure server for a minimum of 20 years.

9 MONITORING AND REGISTRATION

Since the trial uses mostly data collected within the SHCS, data monitoring will be exclusively done centrally. All SHCS collected data will undergo SHCS quality control described in the SCHS protocol (BASEC-Nr. 2023-02080). In brief, high data quality will be ensured by three validations steps. First, the data is collected by trained SHCS study nurses or cohort physicians. Second, the data record is then validated, and if necessary supplemented by local research associates at the responsible SHCS centers. After validation, the data is sent to the SHCS data center at the University of Zurich. Third, at the data center, the data is validated and released for research purposes. Additionally, members of the data center conduct regular quality visits at the centers. For the RETUNE trial, members of the RETUNE study team will monitor recruitment numbers and routinely collected data used for the primary or the secondary endpoints on a weekly basis.

As described in section 4.4, RETUNE will collect additional data only in the intervention group regarding usage and safety of the investigational products. These data will be collected via REDCap and managed by a member of the RETUNE study team (CS). A second member of the RETUNE study team will double check the data entry (AA).

Before recruitment of the recruitment of the first participant at a respective site, the PI will perform an in-person site initiation visit. After follow-up of the last patient, the PI will perform an in-person site closure visit (can be performed remotely).

The trial is registered on the Clinical Trials Registry Platform of the National Institute of Health (NCT06789692) as well as in the Swiss National Clinical trial Portal (BASEC-Nr. 2024-02417).

10. FUNDING / PUBLICATION / DECLARATION OF INTEREST

10.1 Funding

[redacted]

10.2 Declaration of Interests

[redacted]

10.2 Publication and dissemination policy

To pursue a clear communication strategy, we will develop a detailed communication plan with our Participant Advisory Board (see section 4.8). Besides publication in peer-reviewed open-access journals, the results will be shared at national and international conferences (e.g. the annual conference of the Society of Nicotine and Tobacco Research, European AIDS conference, Conference on Retroviruses and Opportunistic Infections), at meetings with other HIV cohorts outside of Switzerland, at the local sites, and in the SHCS newsletter in the form of lay summaries. This newsletter is regularly sent to SHCS participants and is published in German, French, and Italian. To increase accessibility, we may translate the results into English and Spanish.

The sponsor enters and publishes a summary of the trial results in clinicaltrials.gov in accordance with ClinO Art. 65a within one year of completion or discontinuation of the trial. An interruption lasting more than two years is considered a discontinuation of the trial.
For the purpose of publication in the public register the sponsor also ensures that a lay summary of the trial results is entered in BASEC within one year of completion or discontinuation of the trial. The entry is made at least in the national languages of Switzerland in which the study participants were recruited.

11. REFERENCES

1. Hasse B, Ledergerber B, Furrer H, et al. Morbidity and aging in HIV-infected persons: the Swiss HIV cohort study. Clin Infect Dis 2011;53(11):1130-9. (In eng). DOI: 10.1093/cid/cir626.
2. Shah ASV, Stelzle D, Lee KK, et al. Global Burden of Atherosclerotic Cardiovascular Disease in People Living With HIV: Systematic Review and Meta-Analysis. Circulation 2018;138(11):1100-1112. (In eng). DOI: 10.1161/circulationaha.117.033369.
3. Freiberg MS, Chang CC, Kuller LH, et al. HIV infection and the risk of acute myocardial infarction. JAMA Intern Med 2013;173(8):614-22. (In eng). DOI: 10.1001/jamainternmed.2013.3728.
4. So-Armah K, Benjamin LA, Bloomfield GS, et al. HIV and cardiovascular disease. Lancet HIV 2020;7(4):e279-e293. (In eng). DOI: 10.1016/s2352-3018(20)30036-9.
5. Helleberg M, Afzal S, Kronborg G, et al. Mortality attributable to smoking among HIV-1-infected individuals: a nationwide, population-based cohort study. Clin Infect Dis 2013;56(5):727-34. (In eng). DOI: 10.1093/cid/cis933.
6. Clifford GM, Lise M, Franceschi S, et al. Lung cancer in the Swiss HIV Cohort Study: role of smoking, immunodeficiency and pulmonary infection. Br J Cancer 2012;106(3):447-52. (In eng). DOI: 10.1038/bjc.2011.558.
7. Smit M, Brinkman K, Geerlings S, et al. Future challenges for clinical care of an ageing population infected with HIV: a modelling study. Lancet Infect Dis 2015;15(7):810-8. (In eng). DOI: 10.1016/s1473-3099(15)00056-0.
8. Islam FM, Wu J, Jansson J, Wilson DP. Relative risk of cardiovascular disease among people living with HIV: a systematic review and meta-analysis. HIV Med 2012;13(8):453-68. (In eng). DOI: 10.1111/j.1468-1293.2012.00996.x.
9. Feinstein MJ. HIV, Subclinical Cardiovascular Disease, and Clinical Progression: Insights From Immunologic Heterogeneity. Jama 2022;328(10):931-932. (In eng). DOI: 10.1001/jama.2022.15226.
10. Silverberg MJ, Lau B, Achenbach CJ, et al. Cumulative Incidence of Cancer Among Persons With HIV in North America: A Cohort Study. Ann Intern Med 2015;163(7):507-18. (In eng). DOI: 10.7326/m14-2768.
11. Haas CB, Engels EA, Horner MJ, et al. Trends and risk of lung cancer among people living with HIV in the USA: a population-based registry linkage study. Lancet HIV 2022;9(10):e700-e708. (In eng). DOI: 10.1016/s2352-3018(22)00219-3.
12. Weber MSR, Duran Ramirez JJ, Hentzien M, et al. Time Trends in Causes of Death in People With Human Immunodeficiency Virus: Insights From the Swiss HIV Cohort Study. Clinical Infectious Diseases 2024. DOI: 10.1093/cid/ciae014.
13. Scherrer AU, Traytel A, Braun DL, et al. Cohort Profile Update: The Swiss HIV Cohort Study (SHCS). Int J Epidemiol 2022;51(1):33-34j. (In eng). DOI: 10.1093/ije/dyab141.
14. SHCS. (https://www.shcs.ch).
15. Spatial, temporal, and demographic patterns in prevalence of smoking tobacco use and attributable disease burden in 204 countries and territories, 1990-2019: a systematic analysis from the Global Burden of Disease Study 2019. Lancet 2021;397(10292):2337-2360. (In eng). DOI: 10.1016/s0140-6736(21)01169-7.
16. Hemkens LG, Bucher HC. HIV infection and cardiovascular disease. Eur Heart J 2014;35(21):1373-81. (In eng). DOI: 10.1093/eurheartj/eht528.
17. Gryaznov D, Chammartin F, Stoeckle M, et al. Smartphone App and Carbon Monoxide Self-Monitoring Support for Smoking Cessation: A Randomized Controlled Trial Nested into the Swiss HIV Cohort Study. J Acquir Immune Defic Syndr 2020;85(1):e8-e11. (In eng). DOI: 10.1097/qai.0000000000002396.
18. Benowitz NL. Nicotine addiction. N Engl J Med 2010;362(24):2295-303. (In eng). DOI: 10.1056/NEJMra0809890.
19. Gottlieb S, Zeller M. A Nicotine-Focused Framework for Public Health. N Engl J Med 2017;377(12):1111-1114. (In eng). DOI: 10.1056/NEJMp1707409.
20. Jakob J, Joss S, Meier AN, et al. The price of nicotine dependence: A comparison of the cost of nicotine across products in Switzerland, Germany, USA, Sweden, France and the UK, in 2019. Tob Prev Cessat 2022;8:42. (In eng). DOI: 10.18332/tpc/156052.
21. Hartmann-Boyce J, Lindson N, Butler AR, et al. Electronic cigarettes for smoking cessation. Cochrane Database of Systematic Reviews 2022(11). DOI: 10.1002/14651858.CD010216.pub7.
22. National Academies of Sciences E, Medicine, Health, et al. In: Eaton DL, Kwan LY, Stratton K, eds. Public Health Consequences of E-Cigarettes. Washington (DC): National Academies Press (US)

Copyright 2018 by the National Academy of Sciences. All rights reserved.; 2018.

23. Farsalinos KE, Spyrou A, Tsimopoulou K, Stefopoulos C, Romagna G, Voudris V. Nicotine absorption from electronic cigarette use: comparison between first and new-generation devices. Sci Rep 2014;4:4133. (In eng). DOI: 10.1038/srep04133.
24. Buchhalter AR, Acosta MC, Evans SE, Breland AB, Eissenberg T. Tobacco abstinence symptom suppression: the role played by the smoking-related stimuli that are delivered by denicotinized cigarettes. Addiction 2005;100(4):550-9. (In eng). DOI: 10.1111/j.1360-0443.2005.01030.x.
25. Barbeau AM, Burda J, Siegel M. Perceived efficacy of e-cigarettes versus nicotine replacement therapy among successful e-cigarette users: a qualitative approach. Addict Sci Clin Pract 2013;8(1):5. (In eng). DOI: 10.1186/1940-0640-8-5.
26. Notley C, Ward E, Dawkins L, Holland R. The unique contribution of e-cigarettes for tobacco harm reduction in supporting smoking relapse prevention. Harm Reduct J 2018;15(1):31. (In eng). DOI: 10.1186/s12954-018-0237-7.
27. McNeill A, Brose LS, Calder R, Bauld L, Robson D. Evidence review of e-cigarettes and heated tobacco products 2018. A report commissioned by public health England London: Public Health England 2018;6.
28. Lindson N, Butler AR, McRobbie H, et al. Electronic cigarettes for smoking cessation. Cochrane Database Syst Rev 2024;1(1):Cd010216. (In eng). DOI: 10.1002/14651858.CD010216.pub8.
29. Jakob J, Joss S, Meier A, et al. The price of nicotine dependence: A comparison of the cost of nicotine across products in Switzerland, Germany, USA, Sweden, France and the UK, in 2019. Tobacco Prevention & Cessation 2022;8(November):1-5.
30. Lunell E, Fagerström K, Hughes J, Pendrill R. Pharmacokinetic Comparison of a Novel Non-tobacco-Based Nicotine Pouch (ZYN) With Conventional, Tobacco-Based Swedish Snus and American Moist Snuff. Nicotine Tob Res 2020;22(10):1757-1763. (In eng). DOI: 10.1093/ntr/ntaa068.
31. Azzopardi D, Ebajemito J, McEwan M, et al. A randomised study to assess the nicotine pharmacokinetics of an oral nicotine pouch and two nicotine replacement therapy products. Sci Rep 2022;12(1):6949. (In eng). DOI: 10.1038/s41598-022-10544-x.
32. McNeill A, Munafò MR. Reducing harm from tobacco use. Journal of Psychopharmacology 2012;27(1):13-18. DOI: 10.1177/0269881112458731.
33. Azzopardi D, Liu C, Murphy J. Chemical characterization of tobacco-free “modern” oral nicotine pouches and their position on the toxicant and risk continuums. Drug and Chemical Toxicology 2022;45(5):2246-2254. DOI: 10.1080/01480545.2021.1925691.
34. Miller-Holt J, Baskerville-Abraham I, Sakimura M, Fukushima T, Puglisi A, Gafner J. In vitro evaluation of mutagenic, cytotoxic, genotoxic and oral irritation potential of nicotine pouch products. Toxicol Rep 2022;9:1316-1324. (In eng). DOI: 10.1016/j.toxrep.2022.06.008.
35. Mallock N, Schulz T, Malke S, Dreiack N, Laux P, Luch A. Levels of nicotine and tobacco-specific nitrosamines in oral nicotine pouches. Tobacco Control 2022:tobaccocontrol-2022-057280. DOI: 10.1136/tc-2022-057280.
36. Sparrock LS, Phan L, Chen-Sankey J, et al. Nicotine Pouch: Awareness, Beliefs, Use, and Susceptibility among Current Tobacco Users in the United States, 2021. Int J Environ Res Public Health 2023;20(3) (In eng). DOI: 10.3390/ijerph20032050.
37. Le Houezec J. Role of nicotine pharmacokinetics in nicotine addiction and nicotine replacement therapy: a review. Int J Tuberc Lung Dis 2003;7(9):811-9. (In eng).
38. Rigotti NA, Kruse GR, Livingstone-Banks J, Hartmann-Boyce J. Treatment of Tobacco Smoking: A Review. JAMA 2022;327(6):566-577. DOI: 10.1001/jama.2022.0395.
39. Patnode CD, Henderson JT, Coppola EL, Melnikow J, Durbin S, Thomas RG. Interventions for Tobacco Cessation in Adults, Including Pregnant Persons: Updated Evidence Report and Systematic Review for the US Preventive Services Task Force. Jama 2021;325(3):280-298. (In eng). DOI: 10.1001/jama.2020.23541.
40. Hartmann-Boyce J, Chepkin SC, Ye W, Bullen C, Lancaster T. Nicotine replacement therapy versus control for smoking cessation. Cochrane Database Syst Rev 2018;5(5):Cd000146. (In eng). DOI: 10.1002/14651858.CD000146.pub5.
41. Hajek P, Phillips-Waller A, Przulj D, et al. A Randomized Trial of E-Cigarettes versus Nicotine-Replacement Therapy. N Engl J Med 2019;380(7):629-637. (In eng). DOI: 10.1056/NEJMoa1808779.
42. Bullen C, Howe C, Laugesen M, et al. Electronic cigarettes for smoking cessation: a randomised controlled trial. Lancet 2013;382(9905):1629-37. (In eng). DOI: 10.1016/s0140-6736(13)61842-5.
43. Walker N, Parag V, Verbiest M, Laking G, Laugesen M, Bullen C. Nicotine patches used in combination with e-cigarettes (with and without nicotine) for smoking cessation: a pragmatic,

randomised trial. Lancet Respir Med 2020;8(1):54-64. (In eng). DOI: 10.1016/s2213-2600(19)30269-3.
44. Eisenberg MJ, Hébert-Losier A, Windle SB, et al. Effect of e-Cigarettes Plus Counseling vs Counseling Alone on Smoking Cessation: A Randomized Clinical Trial. Jama 2020;324(18):1844-1854. (In eng). DOI: 10.1001/jama.2020.18889.
45. Richter KP, Ellerbeck EF. It's time to change the default for tobacco treatment. Addiction 2015;110(3):381-6. (In eng). DOI: 10.1111/add.12734.
46. Faseru B, Mussulman LM, Nazir N, et al. Use of pre-enrollment randomization and delayed consent to maximize participation in a clinical trial of opt-in versus opt-out tobacco treatment. Subst Abus 2022;43(1):1035-1042. (In eng). DOI: 10.1080/08897077.2022.2060441.
47. Carpenter MJ, Wahlquist AE, Dahne J, et al. Effect of unguided e-cigarette provision on uptake, use, and smoking cessation among adults who smoke in the USA: a naturalistic, randomised, controlled clinical trial. eClinicalMedicine 2023:102142. DOI: https://doi.org/10.1016/j.eclinm.2023.102142.
48. Pisinger C, Vestbo J, Borch-Johnsen K, Jørgensen T. It is possible to help smokers in early motivational stages to quit. The Inter99 study. Prev Med 2005;40(3):278-84. (In eng). DOI: 10.1016/j.ypmed.2004.06.011.
49. Campbell KA, Cooper S, Fahy SJ, et al. 'Opt-out' referrals after identifying pregnant smokers using exhaled air carbon monoxide: impact on engagement with smoking cessation support. Tob Control 2017;26(3):300-306. (In eng). DOI: 10.1136/tobaccocontrol-2015-052662.
50. Murad MH, Asi N, Alsawas M, Alahdab F. New evidence pyramid. Evid Based Med 2016;21(4):125-7. (In eng). DOI: 10.1136/ebmed-2016-110401.
51. Zabor EC, Kaizer AM, Hobbs BP. Randomized Controlled Trials. Chest 2020;158(1s):S79-s87. (In eng). DOI: 10.1016/j.chest.2020.03.013.
52. Relton C, Torgerson D, O'Cathain A, Nicholl J. Rethinking pragmatic randomised controlled trials: introducing the "cohort multiple randomised controlled trial" design. Bmj 2010;340:c1066. (In eng). DOI: 10.1136/bmj.c1066.
53. Young-Afat DA, Verkooijen HA, van Gils CH, et al. Brief Report: Staged-informed Consent in the Cohort Multiple Randomized Controlled Trial Design. Epidemiology 2016;27(3):389-92. (In eng). DOI: 10.1097/ede.0000000000000435.
54. van der Velden JM, Verkooijen HM, Young-Afat DA, et al. The cohort multiple randomized controlled trial design: a valid and efficient alternative to pragmatic trials? Int J Epidemiol 2017;46(1):96-102. (In eng). DOI: 10.1093/ije/dyw050.
55. Couwenberg AM, Burbach JPM, May AM, Berbee M, Intven MPW, Verkooijen HM. The trials within cohorts design facilitated efficient patient enrollment and generalizability in oncology setting. J Clin Epidemiol 2020;120:33-39. (In eng). DOI: 10.1016/j.jclinepi.2019.12.015.
56. Amstutz A, Schönenberger CM, Speich B, et al. Characteristics, consent patterns, and challenges of randomized trials using the Trials within Cohorts (TwiCs) design A scoping review. J Clin Epidemiol 2024:111469. (In eng). DOI: 10.1016/j.jclinepi.2024.111469.
57. Ledgerwood DM, Yskes R. Smoking Cessation for People Living With HIV/AIDS: A Literature Review and Synthesis. Nicotine Tob Res 2016;18(12):2177-2184. (In eng). DOI: 10.1093/ntr/ntw126.
58. Cockerham WC, Hamby BW, Oates GR. The Social Determinants of Chronic Disease. American Journal of Preventive Medicine 2017;52(1, Supplement 1):S5-S12. DOI: https://doi.org/10.1016/j.amepre.2016.09.010.
59. Milcarz M, Polanska K, Bak-Romaniszyn L, Kaleta D. Tobacco Health Risk Awareness among Socially Disadvantaged People—A Crucial Tool for Smoking Cessation. International Journal of Environmental Research and Public Health 2018;15(10):2244. (https://www.mdpi.com/1660-4601/15/10/2244).
60. Courtney RJ, Naicker S, Shakeshaft A, Clare P, Martire KA, Mattick RP. Smoking Cessation among Low-Socioeconomic Status and Disadvantaged Population Groups: A Systematic Review of Research Output. International Journal of Environmental Research and Public Health 2015;12(6):6403-6422. (https://www.mdpi.com/1660-4601/12/6/6403).
61. Moyer TP, Charlson JR, Enger RJ, et al. Simultaneous analysis of nicotine, nicotine metabolites, and tobacco alkaloids in serum or urine by tandem mass spectrometry, with clinically relevant metabolic profiles. Clin Chem 2002;48(9):1460-71. (In eng).
62. Suh-Lailam BB, Haglock-Adler CJ, Carlisle HJ, Ohman T, McMillin GA. Reference interval determination for anabasine: a biomarker of active tobacco use. J Anal Toxicol 2014;38(7):416-20. (In eng). DOI: 10.1093/jat/bku059.

63. Jacob P, 3rd, Hatsukami D, Severson H, Hall S, Yu L, Benowitz NL. Anabasine and anatabine as biomarkers for tobacco use during nicotine replacement therapy. Cancer Epidemiol Biomarkers Prev 2002;11(12):1668-73. (In eng).
64. Auer R, Schoeni A, Humair JP, et al. Electronic Nicotine-Delivery Systems for Smoking Cessation. N Engl J Med 2024;390(7):601-610. (In eng). DOI: 10.1056/NEJMoa2308815.
65. Benowitz NL, Bernert JT, Foulds J, et al. Biochemical Verification of Tobacco Use and Abstinence: 2019 Update. Nicotine Tob Res 2020;22(7):1086-1097. (In eng). DOI: 10.1093/ntr/ntz132.
66. von Weymarn LB, Thomson NM, Donny EC, Hatsukami DK, Murphy SE. Quantitation of the Minor Tobacco Alkaloids Nornicotine, Anatabine, and Anabasine in Smokers' Urine by High Throughput Liquid Chromatography-Mass Spectrometry. Chem Res Toxicol 2016;29(3):390-7. (In eng). DOI: 10.1021/acs.chemrestox.5b00521.
67. Cho ER, Brill IK, Gram IT, Brown PE, Jha P. Smoking Cessation and Short- and Longer-Term Mortality. NEJM Evid 2024;3(3):EVIDoa2300272. (In eng). DOI: 10.1056/EVIDoa2300272.
68. Kahan BC, Hindley J, Edwards M, Cro S, Morris TP. The estimands framework: a primer on the ICH E9(R1) addendum. Bmj 2024;384:e076316. (In eng). DOI: 10.1136/bmj-2023-076316.
69. Gal R, Kessels R, Luijken K, et al. Tailored guidance to apply the Estimand framework to Trials within Cohorts (TwiCs) studies. Global Epidemiology 2024;8:100163. DOI: https://doi.org/10.1016/j.gloepi.2024.100163.
70. West R, Hajek P, Stead L, Stapleton J. Outcome criteria in smoking cessation trials: proposal for a common standard. Addiction 2005;100(3):299-303. (In eng). DOI: 10.1111/j.1360-0443.2004.00995.x.
71. Piper ME, Bullen C, Krishnan-Sarin S, et al. Defining and Measuring Abstinence in Clinical Trials of Smoking Cessation Interventions: An Updated Review. Nicotine Tob Res 2020;22(7):1098-1106. (In eng). DOI: 10.1093/ntr/ntz110.
72. group Sw, collaboration ECr. SCORE2 risk prediction algorithms: new models to estimate 10-year risk of cardiovascular disease in Europe. European Heart Journal 2021;42(25):2439-2454. DOI: 10.1093/eurheartj/ehab309.
73. Coart E, Bamps P, Quinaux E, et al. Minimization in randomized clinical trials. Stat Med 2023;42(28):5285-5311. (In eng). DOI: 10.1002/sim.9916.
74. Jackson T, Pinnock H, Liew SM, et al. Patient and public involvement in research: from tokenistic box ticking to valued team members. BMC Med 2020;18(1):79. (In eng). DOI: 10.1186/s12916-020-01544-7.
75. Organisation SCT. Participation Request for Patient and Public Involvement (PPI) Activities Template. 01.03.2023 (https://www.scto.ch/de/patient-and-public-involvement/ppi-resources.html).
76. Staniszewska S, Brett J, Simera I, et al. GRIPP2 reporting checklists: tools to improve reporting of patient and public involvement in research. Bmj 2017;358:j3453. (In eng). DOI: 10.1136/bmj.j3453.
77. Gal R, Monninkhof EM, van Gils CH, et al. The Trials within Cohorts design faced methodological advantages and disadvantages in the exercise oncology setting. J Clin Epidemiol 2019;113:137-146. (In eng). DOI: 10.1016/j.jclinepi.2019.05.017.
78. Eldridge SM, Lancaster GA, Campbell MJ, et al. Defining Feasibility and Pilot Studies in Preparation for Randomised Controlled Trials: Development of a Conceptual Framework. PLoS One 2016;11(3):e0150205. (In eng). DOI: 10.1371/journal.pone.0150205.
79. Kistin C, Silverstein M. Pilot Studies: A Critical but Potentially Misused Component of Interventional Research. JAMA 2015;314(15):1561-1562. DOI: 10.1001/jama.2015.10962.
80. Avery KN, Williamson PR, Gamble C, et al. Informing efficient randomised controlled trials: exploration of challenges in developing progression criteria for internal pilot studies. BMJ Open 2017;7(2):e013537. (In eng). DOI: 10.1136/bmjopen-2016-013537.
81. StataCorp. Stata Statistical Software: Release 17. (https://www.stata.com).
82. Team RC. R: A language and environment for statistical computing. (https://www.R-project.org/).
83. Kahan BC, Morris TP. Improper analysis of trials randomised using stratified blocks or minimisation. Stat Med 2012;31(4):328-40. (In eng). DOI: 10.1002/sim.4431.
84. Hernández AV, Steyerberg EW, Habbema JD. Covariate adjustment in randomized controlled trials with dichotomous outcomes increases statistical power and reduces sample size requirements. J Clin Epidemiol 2004;57(5):454-60. (In eng). DOI: 10.1016/j.jclinepi.2003.09.014.
85. Unit MCT. Covariate adjustment in trials (https://www.mrcctu.ucl.ac.uk/our-research/methodology/analysis/covariate-adjustment-in-trials/).
86. Hernán MA, Robins JM. Per-Protocol Analyses of Pragmatic Trials. New England Journal of Medicine 2017;377(14):1391-1398. DOI: doi:10.1056/NEJMsm1605385.

87. Martin RM, Donovan JL, Turner EL, et al. Effect of a Low-Intensity PSA-Based Screening Intervention on Prostate Cancer Mortality: The CAP Randomized Clinical Trial. Jama 2018;319(9):883-895. (In eng). DOI: 10.1001/jama.2018.0154.
88. Holme Ø, Løberg M, Kalager M, et al. Effect of flexible sigmoidoscopy screening on colorectal cancer incidence and mortality: a randomized clinical trial. Jama 2014;312(6):606-15. (In eng). DOI: 10.1001/jama.2014.8266.
89. Stuart EA, Perry DF, Le HN, Ialongo NS. Estimating intervention effects of prevention programs: accounting for noncompliance. Prev Sci 2008;9(4):288-98. (In eng). DOI: 10.1007/s11121-008-0104-y.
90. Peugh JL, Strotman D, McGrady M, Rausch J, Kashikar-Zuck S. Beyond intent to treat (ITT): A complier average causal effect (CACE) estimation primer. J Sch Psychol 2017;60:7-24. (In eng). DOI: 10.1016/j.jsp.2015.12.006.
91. Robson E, Kamper SJ, Lee H, et al. Compliance with telephone-based lifestyle weight loss programs improves low back pain but not knee pain outcomes: complier average causal effects analyses of 2 randomised trials. Pain 2022;163(7):e862-e868. (In eng). DOI: 10.1097/j.pain.0000000000002506.
92. Cockayne S, Adamson J, Clarke A, et al. Cohort Randomised Controlled Trial of a Multifaceted Podiatry Intervention for the Prevention of Falls in Older People (The REFORM Trial). PLoS One 2017;12(1):e0168712. (In eng). DOI: 10.1371/journal.pone.0168712.
93. Schandelmaier S, Briel M, Varadhan R, et al. Development of the Instrument to assess the Credibility of Effect Modification Analyses (ICEMAN) in randomized controlled trials and meta-analyses. Cmaj 2020;192(32):E901-e906. (In eng). DOI: 10.1503/cmaj.200077.
94. WMA DECLARATION OF HELSINKI – ETHICAL PRINCIPLES FOR MEDICAL RESEARCH INVOLVING HUMAN SUBJECTS. (https://www.wma.net/policies-post/wma-declaration-of-helsinki-ethical-principles-for-medical-research-involving-human-subjects/).
95. Agency EM. International Conference on Harmonization (ICH) E6(R2) Guideline for Good Clinical Practice. (https://www.ema.europa.eu/en/documents/scientific-guideline/ich-guideline-good-clinical-practice-e6r2-step-5_en.pdf).
96. Confederation TFAotS. Federal Act on Research involving Human Beings. 2022.
97. Council SF. Ordinance on Clinical Trials with the exception of Clinical Trials of Medical Devices. 2022.

Supplementary file

Offer of a menu of different nicotine substitute products to REduce Tobacco Use iN pEople living with HIV (RETUNE): An internal pilot study of a randomized trial

Table of content

APPENDIX 2: SURVEY QUESTIONS 2

APPENDIX 3: R PACKAGES USED FOR THE STATISTICAL ANALYSIS 8

APPENDIX 4: SIDE EFFECTS BY USED PRODUCTS 9

APPENDIX 5: EXPERIENCES WITH E-CIGARETTES (N=24) 10

APPENDIX 6: EXPERIENCES WITH NICOTINE PATCHES (N=9)...11

APPENDIX 7: EXPERIENCES WITH NICOTINE POUCHES (N=4) 12

Appendix 2: Survey questions

Record ID	__________________________________
Please enter the date	__________________________________
Which of the following products have you used during the study? (multiple selection possible)	☐ E-cigarettes<br>☐ Nicotine pouches<br>☐ Nicotine patches<br>☐ I have not used my product so far

AESI e-cigarettes

Have you experienced one of the following side effects while using e-cigarettes? (New or increased symptoms)

- ☐ Nausea
- ☐ Emesis
- ☐ Headache
- ☐ Dizziness
- ☐ Respiratory symptoms (cough, phlegm, wheezing, sore throat)
- ☐ Mouth ulcers
- ☐ Skin reactions
- ☐ Mouth irritations
- ☐ Other
- ☐ None

How often did you experienced nausea?

- ◯ At or after every use
- ◯ At or after some use
- ◯ Only at the initiation of the new product
- ◯ Only once

How often did you experienced emesis?

- ◯ At or after every use
- ◯ At or after some use
- ◯ Only at the initiation of the new product
- ◯ Only once

How often did you experienced headache?

- ◯ At or after every use
- ◯ At or after some use
- ◯ Only at the initiation of the new product
- ◯ Only once

How often did you experienced dizziness?

- ◯ At or after every use
- ◯ At or after some use
- ◯ Only at the initiation of the new product
- ◯ Only once

How often did you experienced respiratory symptoms?

- ◯ At or after every use
- ◯ At or after some use
- ◯ Only at the initiation of the new product
- ◯ Only once

How often did you experienced mouth ulcers?

- ◯ At or after every use
- ◯ At or after some use
- ◯ Only at the initiation of the new product
- ◯ Only once

How often did you experienced skin reactions?

How often did you experienced mouth irritations?

- ◯ At or after every use
- ◯ At or after some use
- ◯ Only at the initiation of the new product
- ◯ Only once

Please describe other side effects of e-cigarette use.

____

AESI nicotine pouches

Have you experienced one of the following side effects while using nicotine pouches? (New or increased symptoms)

- ☐ Nausea
- ☐ Emesis
- ☐ Headache
- ☐ Dizziness
- ☐ Respiratory symptoms (cough, phlegm, wheezing, sore throat)
- ☐ Mouth ulcers
- ☐ Skin reactions
- ☐ Mouth irritations
- ☐ Gingival pain
- ☐ Gingival bleeding
- ☐ Other
- ☐ None

Please describe other side effects of nicotine pouch use.

____

How often did you experienced nausea?

- ○ At or after every use
- ○ At or after some use
- ○ Only at the initiation of the new product
- ○ Only once

How often did you experienced emesis?

- ○ At or after every use
- ○ At or after some use
- ○ Only at the initiation of the new product
- ○ Only once

How often did you experienced headache?

- ○ At or after every use
- ○ At or after some use
- ○ Only at the initiation of the new product
- ○ Only once

How often did you experienced dizziness?

- ○ At or after every use
- ○ At or after some use
- ○ Only at the initiation of the new product
- ○ Only once

How often did you experienced respiratory symptoms?

- ○ At or after every use
- ○ At or after some use
- ○ Only at the initiation of the new product
- ○ Only once

How often did you experienced mouth ulcers?

How often did you experienced skin reactions?	◯ At or after every use<br>◯ At or after some use<br>◯ Only at the initiation of the new product<br>◯ Only once
How often did you experienced mouth irritations?	◯ At or after every use<br>◯ At or after some use<br>◯ Only at the initiation of the new product<br>◯ Only once
How often have you experienced gingival pain?	◯ At or after every use<br>◯ At or after some use<br>◯ Only at the initiation of the new product<br>◯ Only once
How often have you experienced gingival bleeding?	◯ At or after every use<br>◯ At or after some use<br>◯ Only at the initiation of the new product<br>◯ Only once

AESI nicotine patches

Have you experienced one of the following side effects while using nicotine patches? (New or increased symptoms)	☐ Nausea<br>☐ Emesis<br>☐ Headache<br>☐ Dizziness<br>☐ Skin reactions<br>☐ Other<br>☐ None
Please describe other side effects of nicotine patch use:	________________________________
How often did you experienced nausea?	◯ At or after every use<br>◯ At or after some use<br>◯ Only at the initiation of the new product<br>◯ Only once
How often did you experienced emesis?	◯ At or after every use<br>◯ At or after some use<br>◯ Only at the initiation of the new product<br>◯ Only once
How often did you experienced headache?	◯ At or after every use<br>◯ At or after some use<br>◯ Only at the initiation of the new product<br>◯ Only once
How often did you experienced dizziness?	◯ At or after every use<br>◯ At or after some use<br>◯ Only at the initiation of the new product<br>◯ Only once

How often did you experienced skin reactions?

Experience e-cigarette

Question	Response
How did you like the e-cigarette overall?	Not at all (0) Neutral (5) Very much (10)<br>*(Place a mark on the scale above)*
Did you like the flavour of the liquid?	Not at all (0) Neutral (5) Very much (10)<br>*(Place a mark on the scale above)*
Would you like to have another flavour?	○ Yes ○ No
Which one?	______________________________
How was the nicotine concentration of the liquid?	○ Too low ○ Just right<br>○ Too high
How would you rate the handling of the c-cigarette?	○ Very easy ○ Easy<br>○ Neutral ○ Hard ○ Very hard
Did you have enough information regarding the use of the e-cigarette?	○ Yes ○ Rather yes ○ Neutral<br>○ Rather no ○ No
What do you think about the following statement?<br>"E-cigarettes are useful to quit smoking."	○ Strongly agree ○ Agree<br>○ Neutral ○ Disagree<br>○ Strongly disagree

General comments on e-cigarettes:

____

Experience nicotine pouch

Question	Response
How did you like the nicotine pouches overall?	Not at all (0) Neutral (5) Very much (10)<br>*(Place a mark on the scale above)*
How did you like the flavour of the nicotine pouches?	Not at all (0) Neutral (5) Very much (10)<br>*(Place a mark on the scale above)*
Would you like to have an other flavour?	○ Yes ○ No
Which one?	______________________________
How was the nicotine concentration of the nicotine pouches?	○ Too low ○ Just right<br>○ Too high

Did you have enough information regarding the use of the nicotine pouches?	Yes ◯ Rather yes ◯ Neutral<br>Rather no ◯ No
What do you think about the following statement?<br><br>"Nicotine pouches are useful to quit smoking."	◯ Strongly agree ◯ Agree<br>◯ Neutral ◯ Disagree<br>◯ Strongly disagree
General comments on nicotine pouches?	

Experience nicotine patch

How did you like the nicotine patches overall?	Not at all (0) Neutral (5) Very much (10)<br>*(Place a mark on the scale above)*
How was the nicotine concentration of the nicotine patches?	◯ To low ◯ Just right<br>◯ To high
Did you have enough information regarding the use of the nicotine patches?	◯ Yes ◯ Rather yes ◯ Neutral<br>◯ Rather no ◯ No
What do you think about the following statement?<br><br>"Nicotine patches are useful to quit smoking."	◯ Strongly agree ◯ Agree<br>◯ Neutral ◯ Disagree<br>◯ Strongly disagree
What do you think about the following statement?<br><br>"Nicotine patches are easy to handle."	◯ Strongly agree ◯ Agree<br>◯ Neutral ◯ Disagree<br>◯ Strongly disagree
What do you think about the following statement?<br><br>"I would recommend nicotine patches to others to quit smoking tobacco cigarettes."	◯ Strongly agree ◯ Agree<br>◯ Neutral ◯ Disagree<br>◯ Strongly disagree

General comments on nicotine patches

Appendix 3: R packages used for the statistical analysis

Package name	Citation
tidyverse	Wickham H, Averick M, Bryan J, Chang W, McGowan LD, François R, Grolemund G, Hayes A, Henry L, Hester J, Kuhn M, Pedersen TL, Miller E, Bache SM, Müller K, Ooms J, Robinson D, Seidel DP, Spinu V, Takahashi K, Vaughan D, Wilke C, Woo K, Yutani H (2019). “Welcome to the tidyverse.” _Journal of Open Source Software_, *4*(43), 1686. doi:10.21105/joss.01686 <https://doi.org/10.21105/joss.01686>.
table1	Rich B (2025). _table1: Tables of Descriptive Statistics in HTML_. doi:10.32614/CRAN.package.table1 <https://doi.org/10.32614/CRAN.package.table1>, R package version 1.5.1, <https://CRAN.R-project.org/package=table1>.
scales	Wickham H, Pedersen T, Seidel D (2025). _scales: Scale Functions for Visualization_. doi:10.32614/CRAN.package.scales <https://doi.org/10.32614/CRAN.package.scales>, R package version 1.4.0, <https://CRAN.R-project.org/package=scales>.
broom	Robinson D, Hayes A, Couch S (2025). _broom: Convert Statistical Objects into Tidy Tibbles_. doi:10.32614/CRAN.package.broom <https://doi.org/10.32614/CRAN.package.broom>, R package version 1.0.10, <https://CRAN.R-project.org/package=broom>.
gtsummary	Sjoberg DD, Whiting K, Curry M, Lavery JA, Larmarange J. Reproducible summary tables with the gtsummary package. The R Journal 2021;13:570–80. https://doi.org/10.32614/RJ-2021-053.
broom.helpers	Larmarange J, Sjoberg D (2025). _broom.helpers: Helpers for Model Coefficients Tibbles_. doi:10.32614/CRAN.package.broom.helpers <https://doi.org/10.32614/CRAN.package.broom.helpers>, R package version 1.22.0, <https://CRAN.R-project.org/package=broom.helpers>.

R version 4.5.1 (2025-06-13)
Platform: aarch64-apple-darwin20
Running under: macOS Tahoe 26.0.1

Appendix 4: Side effects by used products

Completed the pilot study survey	E-cigarette (n=24)	Nicotine patch (n=9)	Nicotine pouch (n=4)	Total (n=37)
Adverse event of special interest *– no. (%)				
Skin reaction	1/24 (4.2%)	5/9 (55.6%)	0/4 (0%)	6/37 (16.2%)
Respiratory symptoms (cough, phlegm, wheezing, sore throat) #	6/24 (25.0%)	0/9 (0%)	0/4 (0%)	6/28 (21.4%)
Nausea	2/24 (8.3%)	1/9 (11.1%)	0/4 (0%)	3/37 (8.1%)
Headache	2/24 (8.3%)	1/9 (11.1%)	0/4 (0%)	2/37 (5.4%)
Mouth irritation#	1/24 (4.2%)	0/9 (0%)	0/4 (0%)	1/28 (3.6%)
Diarrhea	1/24 (4.2%)	0/9 (0%)	0/4 (0%)	1/37 (2.7%)
Constipation	1/24 (4.2%)	0/9 (0%)	0/4 (0%)	1/37 (2.7%)
Emesis	0/24 (0%)	0/9 (0%)	0/4 (0%)	0/37 (0%)
Dizziness	0/24 (0%)	0/9 (0%)	0/4 (0%)	0/37 (0%)
Mouth ulcers#	0/24 (0%)	0/9 (0%)	0/4 (0%)	0/28 (0%)
Gingival pain#	0/24 (0%)	0/9 (0%)	0/4 (0%)	0/28 (0%)
Gingival bleeding#	0/24 (0%)	0/9 (0%)	0/4 (0%)	0/28 (0%)
No adverse events of special interest – no. (%)	13/24 (54.2%)	3/9 (3.3%)	4/4 (100%)	20/37(54.0%)

* Several answer options per participant possible

Only asked among participants with e-cigarettes and nicotine pouches

Appendix 5: Experiences with e-cigarettes (n=24)

How do you rate the e-cigarette overall?

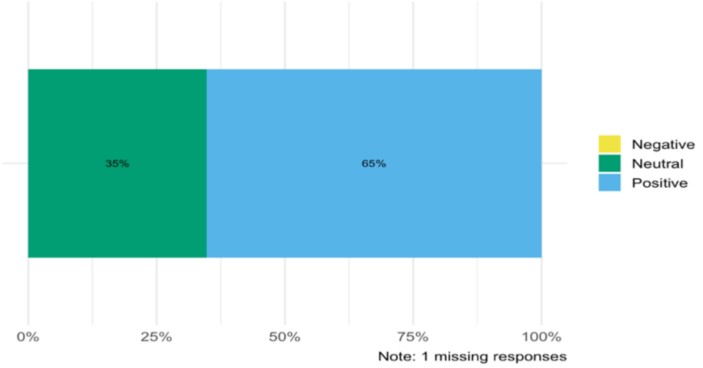

How do you rate the flavor of the liquids overall?

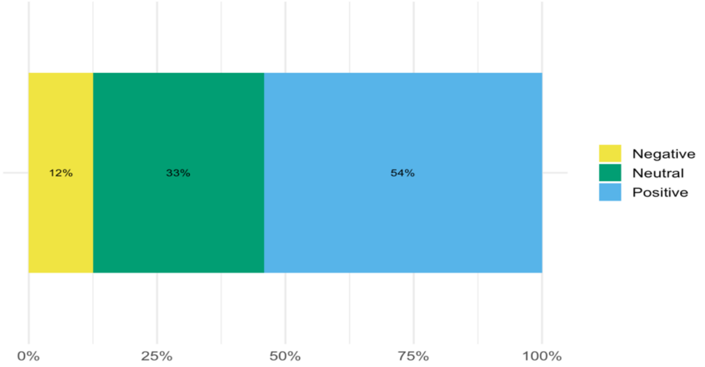

Would you like to have another flavor?

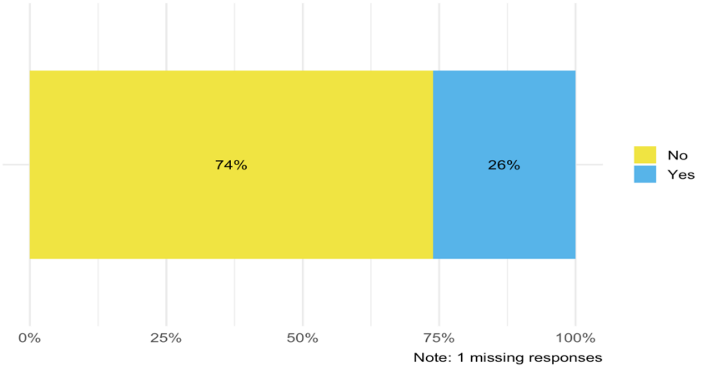

How was the nicotine concentration of the liquid?

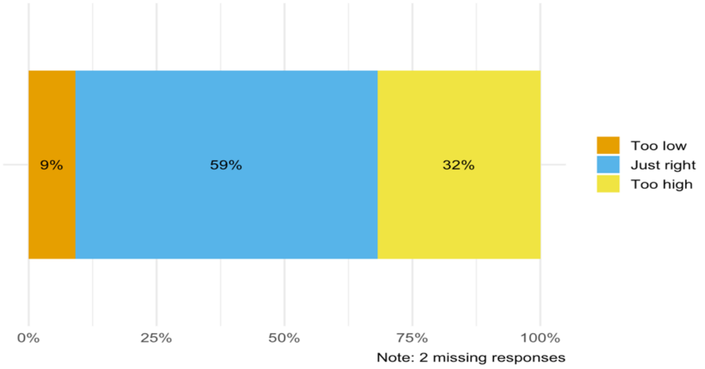

E-cigarettes are useful to quit smoking

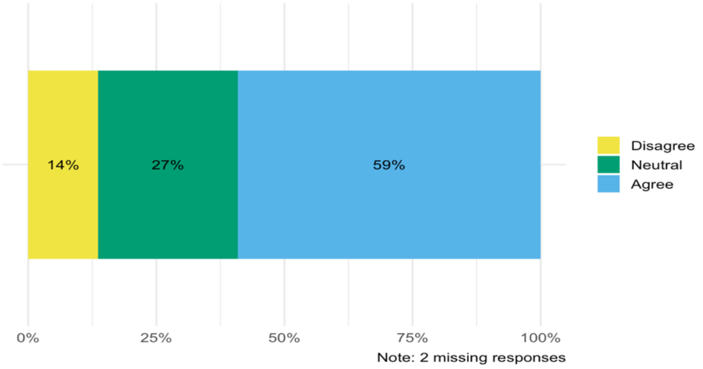

The handling of the e-cigarette was easy

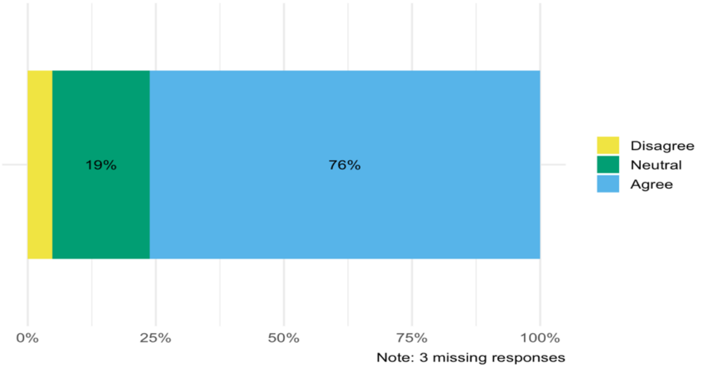

I recieved enough information regarding the use of the e-cigarette

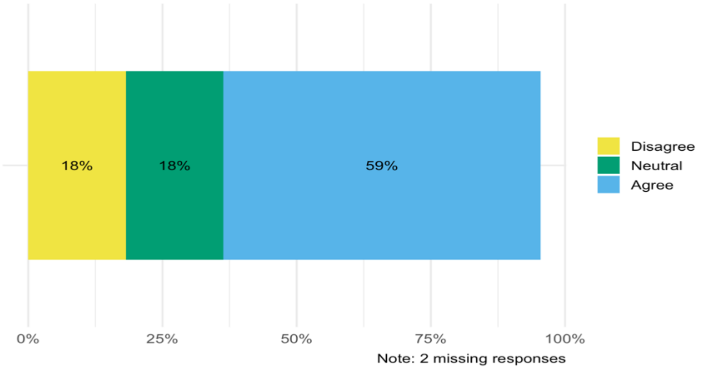

Appendix 6: Experiences with nicotine patches (n=9)

How do you rate the nicotine patch overall?

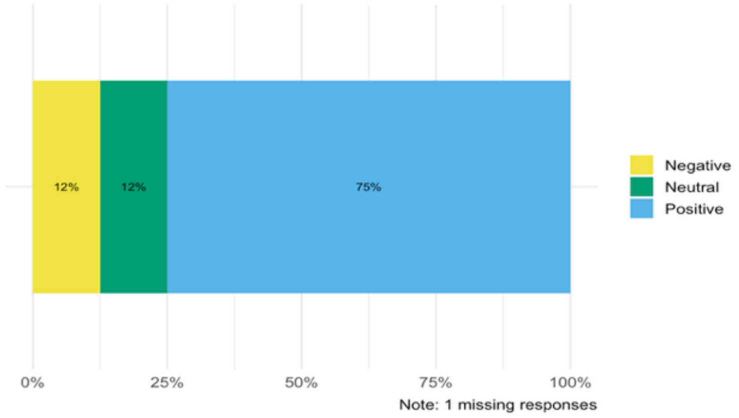

How was the nicotine concentration of the patch?

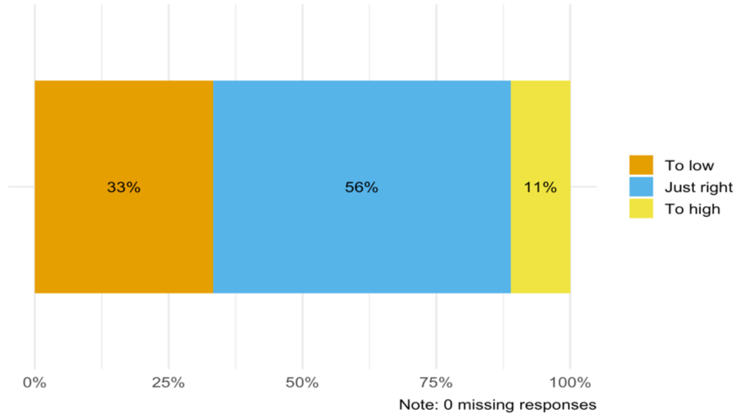

Nicotine patches are useful to quit smoking

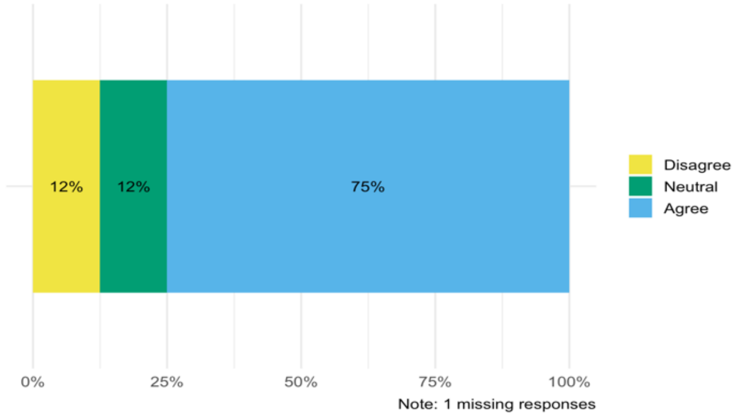

The handling of the nicotine patches was easy

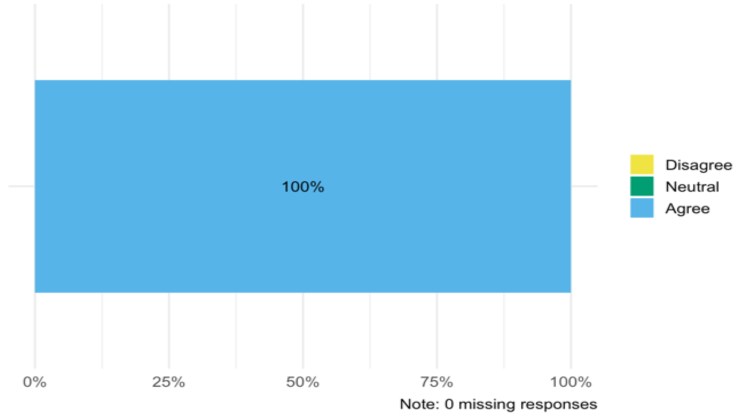

Appendix 7: Experiences with nicotine pouches (n=4)

How do you rate the nicotine pouches overall?

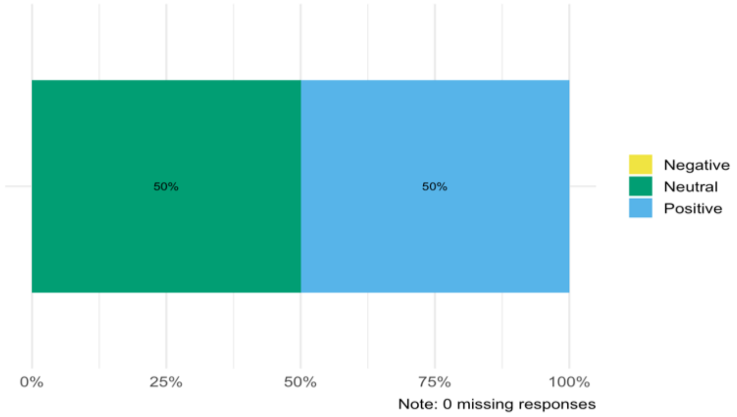

How do you rate the flavor of the pouches overall?

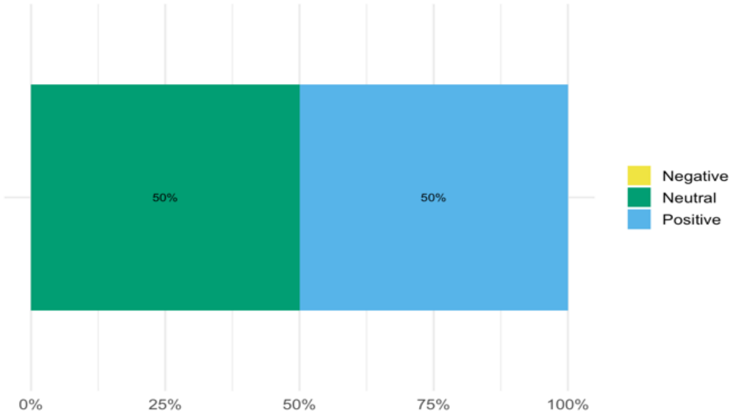

Would you like to have another flavor?

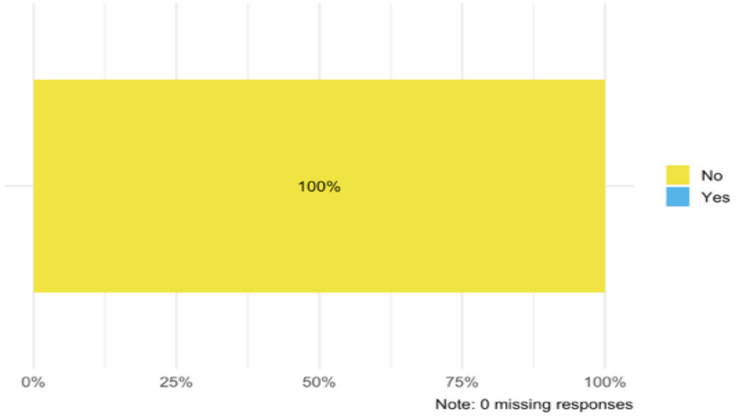

How was the nicotine concentration of the pouch?

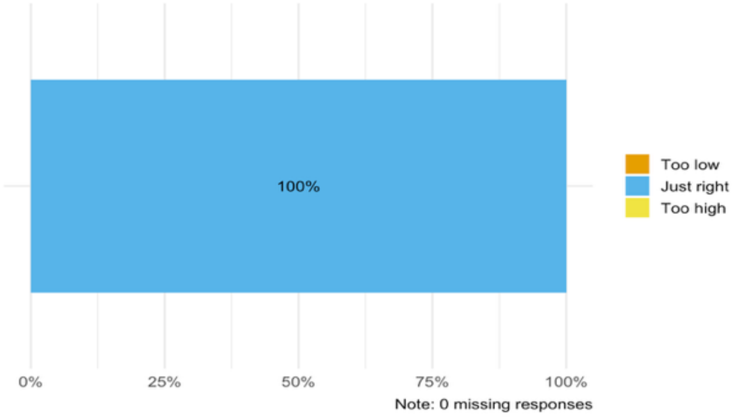

Nicotine pouches are useful to quit smoking

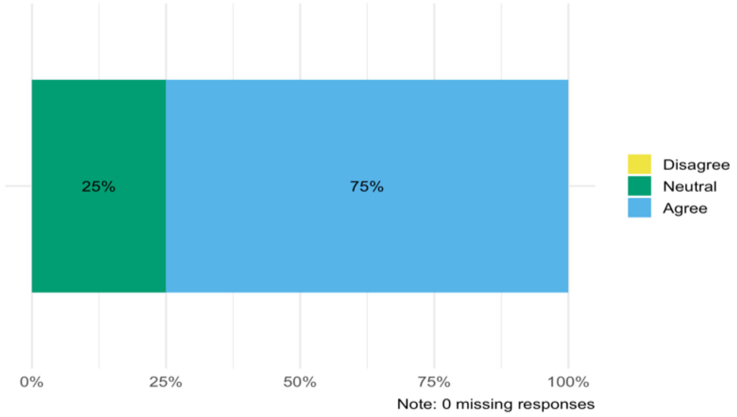

I recieved enough information regarding the use of the nicotine pouches

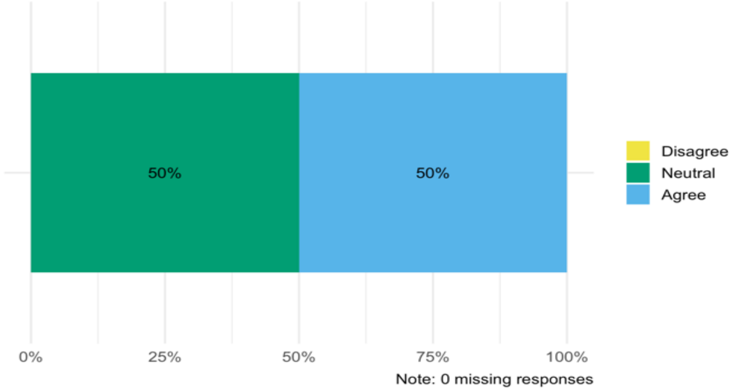

© 2026 Schönenberger C.M. et al.
